# Differentiating care for persons with mild intellectual disability or borderline intellectual functioning: a Delphi study on the opinions of primary and professional caregivers and scientists

**DOI:** 10.1186/s12888-020-2437-4

**Published:** 2020-02-10

**Authors:** Peter J. G. Nouwens, Nienke B. M. Smulders, Petri J. C. M. Embregts, Chijs van Nieuwenhuizen

**Affiliations:** 1 Department of Tranzo, Tilburg School of Social and Behavioral Sciences, Tilburg University, Tilburg, the Netherlands; 2 De Viersprong, Amsterdam, the Netherlands; 3 GGzE Centre for Child & Adolescent Psychiatry, Eindhoven, the Netherlands

**Keywords:** Mild intellectual disability, Borderline intellectual functioning, Support programmes, Differentiation, Delphi

## Abstract

**Background:**

The demand for support for persons with mild intellectual disability or borderline intellectual functioning is growing rapidly. These persons often encounter individual and familial limitations that influence their human functioning, and often have difficulty coping with the demands of modern society. Although in the areas of policy, research and practice, people with mild intellectual disability or borderline intellectual functioning are generally approached as one group, important differences between them have been reported. Current support seems to be both suboptimal and insufficiently differentiated.

**Methods:**

In this Delphi study we aimed to explore the need for appropriate and differentiated support for individuals with mild intellectual disability or borderline intellectual functioning. The study was based on five unique profiles of persons with mild intellectual disability or borderline intellectual functioning that are associated with individual and environmental variables. The opinions of expert primary caregivers, professional caregivers and scientists were analysed for potentially appropriate types of support for each of the five profiles.

**Results:**

A total of 174 statements, divided over the five profiles, were presented to the participants. For 74 statements, consensus was reached between the expert groups. For each profile, these consensual statements represented specific items (e.g. concrete personal goals) and non-specific items (e.g. the attitude towards persons with mild intellectual disability or borderline intellectual functioning, and the coordination of health care) related to the support needs.

**Conclusion:**

This Delphi-based study generated consensual opinions contributing to a more differentiated system of support for individuals with mild intellectual disability or borderline intellectual functioning. Although these findings need additional investigation, they address actions that might enhance the support programmes for these individuals into more personalized support.

## Background

Persons with mild intellectual disability (MID) or borderline intellectual functioning (BIF) often encounter individual and familial limitations that influence their daily functioning, health and quality of life (QoL). For example, they may experience limitations in their social life [1], have fewer friends, and restrictive access to social and leisure activities compared to typically developed individuals [2]. Moreover, many persons with MID or BIF are exposed to a range of negative family conditions, such as divorce, inconsistent parenting, parents with mental health problems, and/or a low financial status [3]. Also, due to the relationship between adverse child, family/contextual factors and mental health problems [4, 5], many persons with MID or BIF suffer from mental health problems [6, 7]. All these findings suggest that persons with MID or BIF are at high risk of experiencing personal and adverse social restrictions in different areas of life, and also underline their vulnerability in a modern and complex society. In the Netherlands, the demand for support for persons with MID or BIF continues to increase, including specialist long-term care [8]. This substantial growth in support needs emphasizes the discrepancy between the capabilities of an individual, the environmental demands [9] and the complexity of modern society. According to Woittiez, Putman, Eggink, and Ras [10], persons with MID or BIF are increasingly unable to function independently without professional support.

In the areas of policy, research and practice, persons with MID and BIF are generally approached as one group. However, important differences have been demonstrated between persons with MID and BIF. For example, Nouwens, Lucas, Embregts, and Van Nieuwenhuizen [3] have shown that persons with BIF encounter more negative individual and social risk factors than persons with MID. Additionally, Emerson, Einfeld, and Stancliffe [11] stated that BIF is associated with poorer mental health in later childhood and adult life. Moreover, there is considerable variation in the lifestyle outcomes (e.g. housing, employment, use of health services, and independent living) within the group of adults with MID or BIF [12].

Using a Latent Class Analysis (LCA), Nouwens, Lucas, Smulders, Embregts, and Van Nieuwenhuizen [13] found discreet classes based on the association between observable variables and revealed patterns of multiple risk factors regarding persons with MID or BIF. Based on an earlier study of the common risk factors for persons with MID or BIF [3], the following environmental and personal variables were included. The category ‘environmental variables’ consisted of two subcategories: 1) family variables and 2) contextual variables; variables were scored as either present or absent. Family variables were: divorce of parents; financial problems of parents; mental health problems of parents; harassment by primary caregiver; sexual abuse by primary caregiver; and inconsistent parenting. The contextual variables were: no informal support from friends and/or family; and difficulty with connecting to peers. The ‘personal variables’ were related to financial problems, daytime activity, alcohol and/or drug addiction, problem behaviour, prison sentence, and mental health problems.

Based on differences in these variables, Nouwens et al. [13] identified five unique profiles of persons with MID or BIF: 1) Persons with mild intellectual disability; 2) Males with problem behaviour; 3) Persons with material hardship and abuse by parents; 4) Male youngsters with problem behaviour and family problems; 5) Persons with addictive problems. These profiles differ with regard to content (i.e. the kind of variables) and complexity and are related to the number of relevant risk factors. Besides the level of cognitive functioning, the accumulation and combination of personal and environmental adverse circumstances seem to be very important for the level of human functioning. Both the presence of and the pattern or association between the variables, result in unique profiles. For example, the difference between Profile 2 and 5 is mainly based on the differences in the environmental variables. Persons in both Profile 2 and Profile 5 have addictive problems. However, individuals in Profile 2 experience fewer adverse environmental conditions as compared to persons in Profile 5. Instead, persons in Profile 5 are often confronted with an accumulation of environmental risk factors (e.g. harassment by primary caregivers, no daytime activity, parents with financial problems, parents with a DSM-IV diagnosis). The unique profiles emphasize the need for a differentiated approach regarding the support for persons with MID or BIF, rather than treating them as one homogeneous group. However, currently, the support for persons with MID or BIF is both suboptimal and insufficiently differentiated [14]. Ideally, appropriate support should be aligned with the individual and contextual characteristics of persons with MID or BIF. According to Thompson et al. [9], adequate support bridges the gap between the environmental demands and the individual capacities, resulting in better human functioning. Regardless of the described differences, people with MID or BIF have much in common [12]. Accessible and individualized supports is crucial for people with MID or BIF. Recognition of this common vulnerability and attention to the associated support needs will do more justice to people with MID or BIF.

The study of Nouwens et al. [13] focused on persons with MID or BIF who rely on the care for ‘people with an intellectual disability’ in the Netherlands. To acquire information on both adequate and better coordination of support, this Delphi study examines the opinions of relevant experts on elements of potentially appropriate care related to each of the five profiles identified by Nouwens et al. [13].

## Methods

A three-round Delphi process [15, 16] was conducted to examine the opinion of primary caregivers, professional caregivers and scientists concerning the support programmes for five subgroups of individuals with MID or BIF (i.e. Profiles 1–5) (Table 1) [13]. The aim was to achieve consensus between three expert groups regarding appropriate support programmes for these five subgroups.

**Table 1:** Description of the five profiles of people with mild intellectual disability or borderline intellectual functioning (*n* = 250)

Profile	*n*	Description	Most prominent problems
Persons with mild intellectual disability (Profile 1)	85	People in the profile ‘Persons with mild intellectual disability’, experience the least individual, family, and contextual problems. Most people in this profile have a mild intellectual disability and in almost half of the cases a comorbid mild autistic disorder is present. The majority has supportive parents. Individuals in Profile 1 experience few personal, environmental and parental (e.g. financial and mental health) problems. In comparison with the other profiles (3, 4, and 5), individuals in Profile 1 receive relatively less informal support.	- Loneliness due to a small social network - The demands of the complex, modern society - Lack of attention from care providers - Lack of social-emotional skills and a restricted self-image
Males with problem behaviour (Profile 2)	51	Individuals in the profile ‘Males with problem behaviour’ show externalising problem behaviour, often have an addiction to alcohol and/or drugs and have parents experiencing difficulties with raising their child. In comparison with the other profiles (3, 4, and 5), individuals in Profile 2 receive relatively less informal support.	- Comorbidity in which the addiction affects the complexity - Relapse in addiction - Restricted self-image and insight
Persons with material hardship and abuse by parents (Profile 3)	47	The profile ‘Persons with material hardship and abuse by parents’ represents mainly women with borderline intellectual functioning who are often a victim of sexual and physical abuse by parents. Some of the people in Profile 3 have a comorbid mood disorder. A small majority of individuals in Profile 3 experience financial problems. The first contact with professional care providers occurs at a relatively high age (M = 23.9 years). Persons in Profile 3 experience relatively more problems with connecting to peers.	- Traumatic and psychiatric problems - Sexual abuse - Negative impact of a vulnerable home environment - Difficulties for professionals to build and maintain a trustful relationship
Male youngsters with problem behaviour and family problems (Profile 4)	37	The profile ‘Male youngsters with problem behaviour and family problems’, includes young men with borderline intellectual functioning displaying externalising problem behaviour, having multiple judicial contacts and who are surrounded by a poor family system. Regarding Profile 4, 100% of the parents are divorced and many parents experienced financial problems; also, the majority of individuals in Profile 4 has been in prison. Compared to the other profiles, persons in Profile 4 received care from the highest number of different healthcare providers (M = 6.5).	- Negative social connections - Family problems - Forensic problems - Behaviour and psychiatric problems - Inappropriate legislation related to the transition to adulthood and forced treatment
Persons with addictive problems (Profile 5)	30	The profile ‘Persons with addictive problems’ is characterised by adults with MID or borderline intellectual functioning having the most serious individual and family problems of all subtypes. All people in this profile have (had) an addiction to alcohol and/or drugs. Most of these people do not have any daytime activities and no permanent residence, are joining the criminal circuit and have been a victim of physical abuse by parents. Compared with the other profiles, individuals in Profile 5 experience relatively major personal financial problems. Persons in Profile 5 experience relatively more problems with connecting to peers.	- Comorbidity and complexity of the problems - Addiction - Lack of structure in daily life - Negative social connections and contact with criminals - Lack of confidence in support and care providers

Adapted from “Identifying classes of persons with mild intellectual disability or borderline intellectual functioning: a latent class analysis,” by P.J.G. Nouwens et al., 2017b, *BMC Psychiatry, 17*, 257

### Recruitment of expert panel

According to Stancliffe et al. [17] relevant information can be directly obtained from people with ID on a variety of topics by the use of self-reports. The outcome of self and proxy-report regarding health-related issues can significantly vary [18]. So, self-reports are important in examining the personal desires, ambitions and needs of individuals with MID or BIF and proxy data cannot validly be considered a substitute. As there is a lack of evidence regarding support and treatment programmes for subgroups of persons with MID or BIF, the Delphi method was executed in the current study. The Delphi method is an appropriate and structured communication process to explore relevant opinions concerning adequate support [15, 16].

The Delphi method however, is an iterative multistage process, using questionnaires, designed to transfer opinions into group consensus [16]. The Delphi method is not adapted to the cognitive and adaptive functioning of persons with MID or BIF and could lead to barriers in communication [17]. Therefore, primary caregivers served as a proxy source for information. For this Delphi study, purposeful sampling was applied to recruit expert participants based on their experience and knowledge [16, 19]. Since a heterogeneous sample results in a higher quality compared to a homogenous sample [16], the study included three different groups of experts from Flanders (Dutch-speaking part of Belgium) and the Netherlands. The expert groups comprised primary caregivers, professional caregivers and scientists.

Primary caregivers were defined as parents, or those individuals responsible by law, with experience in being involved in the upbringing of a person with MID or BIF as a primary source of support. Primary caregivers were contacted: i) by approaching parental foundations for persons with MID or BIF; ii) by asking different health-care organizations for the names of interested primary caregivers; and iii) based on their response to an advertisement about this study in newsletters and websites. Contact information was only shared in cases where the primary caregivers had given permission.

Professional caregivers were individuals with at least 1 year of experience related to the support or treatment of persons with MID or BIF, employed at a health-care organization. These professionals were recruited: i) by consulting different health-care organizations in Belgium and the Netherlands, using a snowball method; and ii) based on their response to an advertisement about this study in newsletters and websites.

Scientists were contacted by searching electronic databases using keywords including ‘mild intellectual disability’, ‘borderline intellectual functioning’, and ‘intellectual disability’, and were recruited using a snowball method. Scientists were asked to nominate colleagues who might be interested in participating in this study, providing that they had research experience related to the target group. To participate, scientists had to have at least one recent (2014–2016) publication related to persons with MID or BIF.

Demographic characteristics of all expert participants are presented in Table 2.

**Table 2:** Demographic characteristics of the participants

	Round 1 (*n* = 30/100%)	Round 2 (*n* = 54/100%)	Round 3 (*n* = 44/100%)
Age in years: mean (*SD)*	49.73 (11.99)	48.85 (11.74)	49.60 (12.45)
Gender ratio (male/female)	10:20	17:36^a^	14:30
Nationality ratio: (Dutch/Belgian)	26:4	46:6^b^	39:5
Background of participants:			
Scientist	10 (33.3%)	13 (24.1%)	11 (25.0%)
Professional caregiver	12 (40%)	21 (38.9%)	19 (43.2%)
Primary caregiver	8 (26.7%)	20 (37.0%)	14 (31.8%)

^a^unknown for 1 participant

^b^unknown for 2 participants

Since the Delphi method does not require representative samples, no additional action was taken to make the three groups representative with respect to the *number* of participants. Instead, for each participant, the *extent of experience* was considered to be of prime importance [16, 20]. Accordingly, based on their experience, the prospective participants were either included or excluded.

### Procedure

An information letter was sent by e-mail to all potential participants. This letter provided information about the background and procedures of the study; the anonymity and confidentiality of all participants were guaranteed. Each of the three rounds involved one questionnaire (which were created using Qualtrics). When experts agreed to participate in the study, a link to the online questionnaires was sent by e-mail. Participants had the opportunity to stop and return at any moment in time to previous answers, without losing the answers they had already filled in. Since poor response is common in Delphi studies [21], a reminder was sent to all participants 1 week and 2 weeks after sending them the questionnaire for each round.

#### Round 1: preparatory round

In Round 1, the questionnaire contained a description of the five profiles of persons with MID or BIF that were identified in an earlier study [13]. Participants were asked to give their opinion on the most appropriate support programmes and most prominent problems per profile (i.e. subgroup), by means of open-ended questions (e.g. *What kind of support or treatment is necessary for a person of subgroup 1? What is missing in the current care for subgroup 1? What is the most prominent problem in Profile 1?*). Open-ended questions were used because of the limited amount of knowledge available on the topic under study [16, 22, 23]. Before the study started, a test panel (consisting of one primary caregiver, one professional caregiver and one scientist) reviewed/checked each questionnaire (for clarity, applicability, etc.) prior to each round. The definitive questionnaire (Round 1) is included as an Additional file 1.

Data on the most appropriate support programmes were analysed using Atlas.ti 7.2 [24]. An inductive approach was used to code the data in a systematic way [25]. For each code, a label was made in which the definition of the code was given. Afterwards, codes were collated into potential categories as a starting point for Round 2. Coding of the data was done by two researchers (N.S. & P.N.). The researchers discussed differences in coding until consensus was reached; based on these discussions, several codes and labels were adjusted. Based on the coding, 174 statements concerning the profiles were constructed. Before Round 2 started, the test panel analysed and reviewed each of these 174 statements (divided over the five profiles).

Per profile, data on the most prominent problems were qualitatively evaluated on face value by each researcher separately. Differences between the researchers were discussed until consensus was reached. Table 1 presents the main problems compiled for each of the five profiles.

#### Round 2: statements about appropriate support

In Round 2, participants were given a description of the profiles and were asked to rate their opinion on the 174 statements using a 5-point Likert scale, ranging from 1 (completely disagree) to 5 (completely agree). The definitive questionnaire of Round 2 is attached as an Additional file 2. The 174 statements were related to potential elements of appropriate support per profile. Since the aim of a Delphi study is to reach consensus between participants, consensus rules were defined prior to analysis of the data. Consensus was reached when an item was rated as ‘agree’ or ‘strongly agree’ by > 80% within each of the three groups (primary caregivers, professional caregivers and scientists). Items rated as ‘agree’ or ‘strongly agree’ by 70–79% in all groups or by > 80% in 1 or 2 expert groups, were rated again in Round 3. Items with consensus levels lower than those described above were rejected and excluded from the study. Of the 174 statements, for 55 statements consensus was reached in Round 2, and 67 statements (divided over the five profiles) proceeded to Round 3 for re-evaluation. Based on the predefined consensus guidelines, 52 statements were eliminated in Round 2.

#### Round 3: re-evaluation in order to achieve consensus

In Round 3, participants received a global summary of the results of Round 2 (i.e. number of statements; number of participants; number of statements for which consensus was reached). Subsequently, the remaining 67 statements were presented to the expert groups. The questionnaire of Round 3 is included as an Additional file 3. The statements in Round 3 consisted of additional information on the level of agreement reached per expert group in Round 2. Based on all the information provided, the participants were asked to re-evaluate each of the 67 statement. A total of 19 statements reached consensus in Round 3. Thus, overall regarding 74 (42.5%) consensus was reached. With regard to the remaining 100 statements (57.7%), no consensus was reached.

### Statistical analysis

Data were analysed with SPSS statistics version 24 on a descriptive level. For each statement in Round 2 and 3, the following variables were calculated: i) level of consensus; ii) mean; ii) standard deviation; iii) mode; iv) range; and iv) importance ranking. All statements that reached consensus in Round 2 or 3 were incorporated in an overall importance ranking.

## Results

In Round 2, a total of 174 statements (divided over the five profiles) was presented to the participants. In 42.52% of these statements (*n* = 74), consensus was reached between the expert groups. These statements were rated as ‘agree’ or ‘strongly agree’ by at least 80% of the participants in each group.

In Round 2, consensus was reached on 55 statements and in Round 3 consensus was reached on 19 statements. Table 3 presents the results, per profile, on the extent of consensus.

**Table 3:** Results of round 2 and 3 for the five Profiles

	Profile 1	Profile 2	Profile 3	Profile 4	Profile 5	Total
No. of statements	27	37	37	35	38	174
Consensus (%)	11 (40.7%)	13 (35.1%)	14 (37.8%)	17 (48.6%)	19 (50.0%)	74 (42.5%)
No consensus (%)	16 (59.3%)	24 (64.9%)	23 (62.2%)	19 (51.4%)	19 (50.0%)	100 (57.7%)

Table 4 in Appendix highlights the top 5 consensus statements per profile attained in Round 2 or 3. A complete overview of all consensus statements (*n* = 74) can be retrieved at the first author. The statements can be divided into specific and non-specific items. Specific items are related to concrete personal goals (e.g. extending social contacts, realizing daytime activities, or work). Non-specific statements are related to the attitude of participants towards individuals with MID or BIF (e.g. positive approach, tenacity in support) or to the coordination of health care (e.g. collaboration between cross-disciplinary professionals and care organizations).

### Profile 1: network and interrelational aspects

Persons in Profile 1 (Persons with mild intellectual disability) are confronted with the least personal and contextual problems. Because individuals in this profile have relatively few friends and are strongly dependent on their family network, attention to resilience and entering/maintaining social contacts was endorsed as being necessary. Many of the statements that were endorsed were related to interrelational aspects, such as cooperation in the assessment of support, and a positive and human approach. Furthermore, in addition to professional support, informal sources of support are appreciated. Of the 27 statements related to Profile 1 (Persons with mild intellectual disability), in Round 2 or 3 consensus was reached between the experts on 11 of these statements. The 11 statements can be divided into four specific (e.g. attention to resilience) and seven non-specific items (e.g. support focused on strengths).

### Profile 2: attention for additional problems and prevention

Besides their MID or BIF all the persons in Profile 2 (Males with problem behaviour) experience additional problems (e.g. behavioural problems or addiction). Prevention of relapse in addiction was the most important issue related to Profile 2. Integrative research focused on multiple additional problems (e.g. behavioural problems), and coordination between various health-care providers is considered necessary. In addition to some relational aspects, many statements were related to the organization of support expressed; for example, in case management, coordination of assistance, early intervention, and aftercare. Of the 37 statements related to Profile 2 (Males with problem behaviour), consensus was achieved on 13 statements: five of these were related to personal goals (e.g. daytime activities) and eight to the attitude or organization of health care (e.g. coordination between relevant sectors of health care).

### Profile 3: integrative approach and durability of support

Persons in Profile 3 (Persons with material hardship and abuse by parents) grow up in families with material hardship and are at relatively high risk of being confronted with inadequate parenting and abuse. Knowledge on all relevant aspects (e.g. intellectual ability, social skills and participation) related to the person was endorsed as being important to conduct appropriate support and treatment programmes. Besides specific forms of support, durability of the support is an important issue expressed in tenacity in seeking and maintaining contact, long-term support, and a permanent core of professionals. Of the 37 statements related to Profile 3 (Persons with material hardship and abuse by parents), in Round 2 or 3 consensus was reached on 14 of these statements. These statements were equally divided between specific (e.g. treatment of mood disorder) and non-specific (e.g. alignment between different health-care providers) items.

### Profile 4: safety and development

Persons in Profile 4 (Male youngsters with problem behaviour and family problems) are not only confronted with relatively more personal problems, but also grow up in a household with multiple family issues. For example, all the parents of persons in this profile are divorced, and in almost every family one of the parents has a mental health problem. An emphasis on safety, an assessment of potential risks and a supporting environment were endorsed as important. Cooperation between different health-care providers and case management was considered necessary. Persons in Profile 4 have the lowest average age. A relatively large number of statements with an educational and developmental character (e.g. perspective for the future, support in education, guidance in independent living) were endorsed. For Profile 4 (Male youngsters with problem behaviour and family problems), of the 35 statements that emerged, consensus was reached between the expert groups for 17 of them. Of these statements, 11 were related to concrete personal goals (e.g. independent living).

### Profile 5: basic facilities and an integrative approach

Persons in Profile 5 (Persons with addictive problems) experience an accumulation of personal and environmental problems. They have no day activities, are addicted, and their parenting is very limited. Within this profile the intellectual disability of the person involved has to be taken into account, besides paying attention to multiple additional problems. Furthermore, relatively more statements are related to basic facilities such as a safe home, a day structure, and the basic necessities of life. Endurance and tenacity, a positive approach, and attention to the motivation to allow support were endorsed. Finally, great importance was attached to the coordination and organization of support. The highest number of statements (*n* = 38) was formulated for Profile 5 (Persons with addictive problems) and consensus between the expert groups was reached for 50% of these statements. In total, 13 statements were related to personal goals.

## Discussion

Support and treatment programmes for people with MID or BIF are suboptimal and more differentiation is required in the services provided to these individuals [14]. The study of Nouwens et al. [13] revealed not only differences between people with MID or BIF, but also differences in personal and environmental characteristics between the identified profiles. Besides the level of cognitive functioning, the accumulation and combination of personal and environmental adverse circumstances seem to be very important for the level of human functioning. The presence or absence of these factors seems to determine, to a large extent, the heterogeneity in the group. Support should be adequately aligned with personal and environmental circumstances, as well as with the level of cognitive functioning. Therefore, the present study explored the opinion of expert groups (primary caregivers, professional caregivers, and scientists) regarding the elements of support and treatment for persons with MID or BIF per profile, as identified by Nouwens et al. [13].

### Profile-specific issues

Several profile-specific issues were revealed. With regard to ‘Persons with mild intellectual disability’ (Profile 1), attention to resilience was endorsed as the most important element. Resilience refers to the resistance to environmental risks or overcoming adversity [26]. According to Emerson [26], resilience is related to individual (e.g. intelligence, problem-solving skills) and contextual characteristics (e.g. family characteristics). For the majority of individuals in Profile 1, supportive relatives are very important; however, due to a relatively small network, these individuals are also very dependent on these relatives. Attention to resilience should enhance the autonomy of these individuals and was indicated as being crucial. Furthermore, in line with Hermsen et al. [27], and Embregts [28], professional care should be experienced as ‘natural’ and should focus on the interpersonal relationship. Due to a relatively small number of problems in Profile 1, the emphasis in support may be more on the relationships than on the actual intervention.

Regarding ‘Males with problem behaviour’ (Profile 2), attention to and prevention from relapse in addiction and aftercare subsequent to treatment were indicated as important profile-specific issues. Addiction is a common problem in relation to Profile 2. According to Bailey et al. [29], 40–60% of drug misusers relapse and require multiple long-term treatment or support programmes. This endorses the opinions of the experts involved in the present study.

Attention to mood disorder, if present, was endorsed as vital for ‘Persons with material hardship and abuse by parents’ (Profile 3). As stated by Lunsky [30], mood disorders might be caused by sexual abuse from primary caregivers. To prevent sexual abuse or maltreatment, individuals should be protected against persons with wrong intentions in their environment. Furthermore, tenacity in seeking and maintaining contact was endorsed as being necessary. Individuals in Profile 3 were relatively old at the moment of their first contact with a health-care provider [13]. A childhood in a family with numerous risk factors, combined with the relatively restricted and delayed attention of health-care providers, endorses the experts’ opinions. Additionally, because ‘attachment insecurity’ in childhood is strongly influenced by the maltreating behaviour of parents, this could result in attachment-avoiding strategies of persons with MID or BIF.

‘Male youngsters with problem behaviour and family problems’ (Profile 4) experienced many family problems and were often surrounded by a poor family system. Furthermore, 100% of the parents involved were divorced [13]. Not surprisingly, the experts endorsed the need for a safety net of professionals in case of a person’s relapse and attention to a safe home, as two important profile-specific items. A second issue for Profile 4 was attention to independent living and a future perspective. Because individuals in Profile 4 are relatively young compared with those in the other profiles [13], a meaningful future perspective is important. Additionally, the experts endorsed that the treatment and support should remain the same when the individual reaches 18 years of age. According to Rot [31] and Van Rijn [32], youngsters with MID or BIF experience problems with the transition to adulthood. Individuals with MID or BIF are often characterized by a discrepancy between their developmental age and biological age. The sudden transition to legal adulthood and the related responsibilities, combined with the vulnerabilities of these individuals, might cause major problems.

‘Persons with addictive problems’ (Profile 5) had the highest number of statements that reached consensus (*n* = 19) and had the most profile-specific issues (*n* = 7). Profile 5 is characterized by a broad spectrum of complex problems, which can exist simultaneously [13]. The role of the intellectual disability was almost unanimously endorsed as essential for the treatment and support for individuals in Profile 5. According to Wieland and Zitman [33], recognition and acknowledgement of the intellectual disability could enhance the quality of the mental health care. Surprisingly, the treatment of addiction problems was not mentioned as a specific point of attention. As stated by Simpson [34], in the support for people with an intellectual disability, addiction is commonly viewed as a simple behavioural problem. Nevertheless, the importance of assessment and effective treatment interventions for people with an intellectual disability in combination with addiction problems has been reported [35]. In view of the broad spectrum of complex problems present in this profile, the following issues were endorsed as important by the experts: acceptance of support; endurance and tenacity in treatment and support; attention to criminal and problem behaviour; a clear daily structure; and the development of self-confidence and a positive self-image. Lastly, parenting support was labelled as valuable. Profile 5 is characterized by a relatively high number of individuals who are already a parent [13]. As stated by Feldman et al. [36], children of parents with an intellectual disability are at high risk of neglect and the development of behavioural problems.

### Overarching key issues

Despite differences between the five profiles, several overarching and recurring key issues in all profiles were endorsed by the expert groups. First, in line with Shogren, Wehmeyer, Buchanan, and Lopez [37], the participating experts underlined the importance of an approach focused on the capabilities and strengths of the persons with MID or BIF. The intellectual disability of individuals with MID or BIF is regularly identified by restrictions in cognitive and social adaptive skills. Moreover, support and treatment programmes for persons with MID or BIF are mainly oriented towards problems and disabilities [3]. More emphasis on the competences and strengths of people with MID or BIF is important to enhance their participation in society and quality of life [12, 37].

Second, the coordination and cooperation between different health-care providers is a recurring issue in the opinion of the expert groups. Effective collaboration between different health-care providers is essential in view of the presence of multiple problems; however, collaboration between different health-care providers or disciplines is sometimes inadequate. As a result, the provision of support or treatment programmes is often incomplete or fragmented [14]. As suggested by Dosen [38], the present expert groups also endorsed that appropriate and differentiated care should depend on an integrative assessment of all relevant aspects related to the demands of support. An incomplete view regarding a disability or an individual could obstruct the collaboration and coordination between health-care providers, potentially resulting in suboptimal health-care programmes.

Third, early intervention to prevent the emergence of major problems was a recurring key issue for all profiles (excluding Profile 1). Nouwens et al. [13], and Embregts et al. [39], noted the relatively high average age at which individuals with MID or BIF received support. According to Allen et al. problems that are noticed (too) late, could become difficult to treat or resistant to training [40].

Fourth, persons with an intellectual disability seem to have fewer social contacts in terms of relatives (and/or friends). In almost all profiles, individuals experienced difficulties regarding entering and maintaining a positive or supportive social network [13]. However, a supportive social network is endorsed as vital for all profiles (apart from Profile 2). According to Reinders [41], a positive and supportive social network (e.g. friends) can be seen as a main influencing factor that enhances the quality of life. Moreover, as stated by McGillivray and McCabe [42], the closeness in these social relationships may be more important than the frequency of contact. Furthermore, negative or fraught relationships had adverse emotional outcomes [42, 43].

Fifth, fewer people with an intellectual disability are employed compared to people without such a disability [44]. Therefore, participation and guidance in daytime activities is a prominent issue recurring in all profiles (excluding Profile 1). According to Schalock et al. [45], quality of life is influenced by a useful daytime activity (e.g. work). In addition, employment contributes to belonging and feeling appreciated [46, 47].

Lastly, people with a MID or BIF face similar and significant challenges in a complex society. A need for professional support can arise when there is a discrepancy between their personal qualities and the demands from the environment. However, necessary support is not always guaranteed, and problems are often noticed too late. Problems can have been present for years before they are recognized. More attention to prevention and early signalling of problems of persons with MID or BIF and their families by mainstream services is needed [48]. The current study emphasizes that underlying limiting conditions of people with MID or BIF are more crucial in determining the appropriate support than solely using cut-off points regarding IQ and adaptive behaviour. People with MID or BIF in the Netherlands have often been approached as one group in policy, research and practice. The current support seems suboptimal and insufficiently differentiated [14]. Appropriate support needs to be aligned with the specific personal and contextual characteristics of individuals with MID or BIF. The support should not only be problem orientated but must also focus on the capabilities and preferences of the persons with MID or BIF [37].

People with MID or BIF are relatively often confronted with additional problems (e.g. mental health problems and addiction). Specialized care is required that incorporates all the relevant aspects and should be based on a cross disciplinary collaboration between different kinds of healthcare providers (e.g. specialised ID services, mental health care and specialised addiction services). In all cases, this care should be aligned with the specific information processing level, the limitations in the regulation and cognitive functions [49]. Despite the increased attention for people with MID or BIF in the Netherlands there is still a long way to go in realising adequate and personal support.

### Study limitations

Although we aimed for a high level of accuracy, some limitations need to be addressed. First, the identified profiles consisted of persons with MID or BIF who rely on the care for ‘people with an intellectual disability’ in the Netherlands. Accordingly, the study concerns a clinical subpopulation and is, therefore, not representative of the entire population of individuals with MID or BIF in the Netherlands. Consequently, no generalizations can be made to the whole population. Second, besides the feedback on the results provided in Round 3, multiple factors could cause a change (increase/decrease) in the levels of agreement. As this study was performed blind, little demographic information was collected on the individual participants and such details could not be acquired after the study; therefore, there might be differences in the composition of the expert groups that are (almost) impossible to detect. Second, participants were asked to rank their opinion regarding statements that concerned all five profiles; however, some participants might lack experience with the entire spectrum, implying that they had to give/rank their opinion on a relatively unfamiliar area. Third, the current study was based on a Delphi method that addressed the opinions of primary caregivers, professional caregivers and scientists on the elements of support and treatment for individuals with MID or BIF per distinguished profile. However, because the opinions of these individuals that actually experienced this support system were not solicited, the perspective of the recipients of treatment was absent. Although persons with MID or BIF are able to express their needs for support, the Delphi method is not adapted to their capabilities. Future research should also incorporate the perspective of persons with MID or BIF. Fourth, we used an LCA resulting in five unique profiles of people with MID or BIF who rely on the care for ‘people with an intellectual disability’. LCA identifies unobservable subgroups within a population. These profiles allow scientists to better understand the impact of exposure to patterns of multiple risks. Besides a better understanding, these profiles also provide insight into the support needs and offer direction to the support programmes, since all the potential risk factors are included. However, the use of profiles should never lead to less attention to the idiographic and individual wishes, goals and support needs of persons with MID or BIF. Conceptualizing support and the support needs of persons with MID or BIF always starts with identifying the desired life, experiences and goals, and always needs to be personalized [9]. Finally, the small sample size precludes generalization to a larger or different population.

### Future directions

According to the United Nations’ Convention on the Rights of Persons with Disabilities [50], persons with MID or BIF have the right to live independently and have legitimate access to personalized support if they cannot realize this on their own. The authors endorse these rights and hope that this study might contribute to a better quality of life for persons with MID or BIF. We have conducted research on the opinion of experts about appropriate support. The consensual opinions of the experts might contribute to more differentiated support that better meets the needs of persons with MID or BIF. Relatively more persons with MID or BIF are confronted with multiple related risks that can impede the achievement of their personal goals in life. Attention to their living conditions and support needs that addresses the multidimensionality of life is important for effective support, to help realize the personal goals of persons with MID or BIF, and to enhance their quality of life. Appropriate support can bridge the gap between the individual capabilities and contextual demands, when the personal goals and needs and the multidimensionality of life are taken into account, resulting in human functioning outcomes [9].

## Conclusions

This Delphi-based study generated consensual opinions that might contribute to a more personalized support system for individuals with MID or BIF. Nevertheless, the findings only address actions that might be appropriate. To achieve improved support programmes, these opinions need to be explored in more detail. The actions should be operationalized and incorporated in the specific service programmes per profile. Examining the effectiveness of this additional actions is of great importance. Both subjective (e.g. self-reports) and objective measures are needed to assess the effectiveness. People with MID or BIF should be directly involved in the assessment of their QoL, for example by assessing with instruments that are adjusted to the communication level of persons with ID. For the evaluations of the service programmes the use of objective parameters for QoL are recommended [51]. Based on the results of these measurements, the actions can be improved in a plan-do-check-act (PDCA) cycle resulting in a continues improvement process.

### Supplementary information

**Additional file 1.** Questionnaire: Round 1 Delphi study.

**Additional file 2.** Questionnaire: Round 2 Delphi study.

**Additional file 3.** Questionnaire: Round 3 Delphi study.

## Data Availability

The questionnaires are included as attached files. The data sets used and analysed are available from the corresponding author on reasonable request.

## Appendix

**Table 4:** Top five consensus statements per profile (Round 2 & 3)

Rank	Item text	Overall						Scientists	Professional caregivers	Primary caregivers
		Consensus in round	S/N-S^a^	Level of agreement (%)	Mean (SD)	Mode^b^	Range	Level of agreement (%)	Level of agreement (%)	Level of agreement (%)
*Profile: Persons with mild intellectual disability*										
1	Attention to their resilience is important.	3	S	97.8	4.18 (.45)	4	2	90.9	100	100
2	Treatment and support must focus on the possibilities of the person.	2	N-S	96.3	4.05 (.58)	5	2	92.3	95.2	100
3	The intensity of treatment and support can easily be adjusted (a lot if necessary, a little if things are going well).	2	N-S	94.4	4.44 (.60)	5	2	92.3	90.5	100
4	Support from own network (family, friends, acquaintances) is important.	2	N-S	94.4	4.24 (.55)	4	2	84.7	95.2	100
5	Support with regard to entering into and maintaining social contacts is necessary.	3	S	93.2	4.14 (.60)	4	3	90.9	89.5	100
*Profile: Males with problem behaviour*										
1	A relapse in addiction must be prevented.	3	S	97.7	4.23 (.48)	4	2	100	94.7	100
2	The treatment and support must focus on the possibilities of the person.	2	N-S	96.3	4.56 (.57)	5	2	92.3	95.2	100
3	Appropriate research on additional problems besides the intellectual disability is required.	2	S	96.2	4.44 (.57)	4	2	100	90.4	100
4	Coordination of the various sectors in healthcare is necessary.	2	N-S	94.4	4.43 (.60)	5	2	100	95.2	89.5
5	Support with regard to finding and keeping daytime activities/ work is very important.	3	S	93.2	4.30 (.59)	4	2	100	89.5	92.9
*Profile: Persons with material hardship and abuse by parents*										
1	Knowledge and overview of all aspects related to the person (e.g. intellectual ability, social skills, participation in society) are necessary to achieve an optimal treatment and support program.	2	S	98.2	4.50 (.54)	5	2	100	100	94.7
2	Attention to the qualities of the individual is required.	2	N-S	98.1	4.48 (.54)	5	2	92.3	100	100
3	Tenacity in seeking and maintaining contact is necessary.	2	N-S	96.3	4.41 (.57)	4	2	92.3	100	94.7
4	Early support is needed to prevent the escalation of problems.	2	N-S	96.2	4.44 (.57)	4	2	92.3	100	94.8
5	Guidance on finances or debts is necessary.	3	S	95.4	4.32 (.64)	4	3	100	89.5	100
*Profile: Male youngsters with problem behaviour and family problems*										
1	A safety net with people in case of relapse is important	2	S	98.1	4.53 (.54)	5	2	92.3	100	100
2	Guidance in independent living is indispensable.	3	S	97.8	4.25 (.49)	4	2	100	94.7	100
3	Cooperation between the different healthcare providers who are involved with the person is necessary.	2	N-S	96.2	4.54 (.58)	5	2	100	95.2	89.5
4	Attention to a safe (home) environment is required.	2	S	96.2	4.49 (.58)	5	2	92.3	95.2	100
5	A perspective must be offered for the future.	3	S	95.5	4.30 (.63)	4	3	100	94.7	92.9
Profile: Persons with addictive problems										
1	The intellectual disability must be taken into account during treatment and support.	2	S	98.1	4.58 (.54)	5	2	92.3	100	100
2	Endurance and tenacity in treatment and support are required.	2	N-S	96.3	4.30 (.54)	4	2	92.3	95.2	100
3	Cooperation between the different healthcare organisations is very important.	2	N-S	96.2	4.47 (.64)	5	3	100	100	89.9
4	A positive approach is necessary.	2	N-S	94.4	4.42 (.60)	4	2	92.3	100	89.5
5	Long-term support must be provided.	2	N-S	94.4	4.40 (.66)	4	3	92.4	95.2	94.7

^a^*S* Specific, *N-S* Non-specific, ^b^ 4 - agree; 5 – strongly agree

## Abbreviations

BIF: Borderline intellectual functioning
DSM-IV: Diagnostic and Statistical Manual of Mental Disorders version 4
ID: Intellectual disability
LCA: Latent Class Analysis
MID: Mild intellectual disability
QoL: Quality of life
SPSS: Statistical Package for the Social Sciences
