# Supplementary material for: Differentiating care for persons with mild intellectual disability or borderline intellectual functioning: a Delphi study on the opinions of primary and professional caregivers and scientists
Source: BMC Psychiatry. 2020 Feb 10;20:57. doi: 10.1186/s12888-020-2437-4 (PMC7008567; doi:10.1186/s12888-020-2437-4)
Supplement: Supplementary file 1 — Additional file 1. Questionnaire: Round 1 Delphi study. [file 12888_2020_2437_MOESM1_ESM.docx]

**Questionnaire: Round 1 Delphi study**

**Introduction**

The goal of this Delphi study is to reach consensus between expert groups regarding the appropriate care for persons with mild intellectual disability (MID) or borderline intellectual function (BIF).
Using this Delphi study, we aim to investigate what kind of support i) parents of individuals with MID or BIF, ii) researchers and iii) professional caregivers would endorse per profile.

The research consists of three rounds.

Round 1 provides a brief description of the 5 profiles of persons with MID or BIF; these profiles are based on scientific research. We would like to ask you to answer 6 open-ended questions regarding your opinion about the appropriate support for persons with MID or BIF, as described in the profiles.

Please note, there are no right or wrong answers, we are only interested in your own insights and ideas. Completing the entire questionnaire will probably take about 60-90 minutes.

Thank you in advance for your cooperation!

With kind regards,

Peter Nouwens, Nienke Smulders, Petri Embregts and Chijs van Nieuwenhuizen

**Profile 1: Persons with mild intellectual disability**

The population included in this profile mainly consists of men with an average age of 27 years. Most of them have a mild intellectual disability; sometimes, they have a mild form of autism. Almost all persons in profile 1 have a day activity or work. However, because they have difficulty in maintaining social contacts, they have few friends. Their parents are competent in parenting and, in most cases, provide social/emotional support.

1. In your opinion, what kind of support or treatment is required for persons in this profile?
2. What is your reason(s) for choosing this kind of support or treatment?
3. What is the main problem for persons in this profile?
4. In your opinion, what difficulties are related to the problems of persons in profile 1?
5. What do you miss in the actual support for persons in profile 1?
6. Do you have any additional comments/ideas about profile 1?

**Profile 2: Males with problem behaviour**

This profile consists mainly of men with borderline intellectual functioning and with behavioural problems; their average age is 25 years. Individuals in this profile have often been addicted to alcohol and/or drugs. Most of these persons experience difficulty in maintaining friendships. In the past, they may have had contact with the police and/or judicial authorities. Most have some form of day activity or work. Their parents are generally emotionally supportive, but had difficulty raising their child. Parents received almost no help in raising their child from family or friends. Some mothers of individuals in this profile have their own mental health problems.

1. In your opinion, what kind of support or treatment is required for persons in this profile?
2. What is your reason(s) for choosing this kind of support or treatment?
3. What is the main problem for persons in this profile?
4. In your opinion, what difficulties are related to the problems of persons in profile 2?
5. What do you miss in the actual support for persons in profile 2?
6. Do you have any additional comments/ideas about profile 2?

**Profile 3: Persons with material hardship and abuse by parents**

Most persons in this profile are women with borderline intellectual functioning; their average age is 30 years. Some of the persons in this profile may have a mood disorder; furthermore, they often have debts. Most people in this profile have difficulties in maintaining friendships. A relatively large proportion of this group have been subjected to sexual and/or physical abuse by their parents; moreover, their parents were inconsistent in their upbringing style. The brothers and sisters of these individuals often have psychological problems themselves.

1. In your opinion, what kind of support or treatment is required for persons in this profile?
2. What is your reason(s) for choosing this kind of support or treatment?
3. What is the main problem for persons in this profile?
4. In your opinion, what difficulties are related to the problems of persons in profile 3?
5. What do you miss in the actual support for persons in profile 3?
6. Do you have any additional comments/ideas about profile 3?

**Profile 4: Male youngsters with problem behaviour and family problems**

This profile mainly consists of young men with borderline intellectual functioning; in this profile, persons with mild intellectual disability or borderline intellectual functioning have an average age of 19 years. All persons in this profile show behavioural problems. A relatively large proportion has been in contact with the police/judicial authorities, or has been in prison. All persons in this profile go to school. Although most of them have friends, they are surrounded by a vulnerable family system. All their parents are divorced and often also have financial problems; however, these parents receive a relatively large amount of informal support that might help their parenting.

1. In your opinion, what kind of support or treatment is required for persons in this profile?
2. What is your reason(s) for choosing this kind of support or treatment?
3. What is the main problem for persons in this profile?
4. In your opinion, what difficulties are related to the problems of persons in profile 4?
5. What do you miss in the actual support for persons in profile 4?
6. Do you have any additional comments/ideas about profile 4?

**Profile 5:** **Persons with addictive problems**

Most people in this profile have borderline intellectual functioning and behavioural problems, and all are addicted to alcohol and/or drugs. This profile has an almost equal distribution between men and women; the average age is 28 years. More than half of these persons have no permanent residence or home. No-one in this profile has any form of daytime activities or work. Also, most of them have difficulty establishing relationships with their peers. A relatively large proportion has contact with the criminal world and/or contact with the police and judicial authorities. Debts are common. Most persons in this profile have a partner, and some have one or more children. Generally, the family in which they were raised had various problems; most of their parents are divorced. Many persons in this group were abused by their parents. In addition, many of the parents had debts, mental health problems, and also had difficulty raising their children in a consistent and appropriate way.

1. In your opinion, what kind of support or treatment is required for persons in this profile?
2. What is your reason(s) for choosing this kind of support or treatment?
3. What is the main problem for persons in this profile?
4. In your opinion, what difficulties are related to the problems of persons in profile 5?
5. What do you miss in the actual support for persons in profile 5?
6. Do you have any additional comments/ideas about profile 5?
