# Supplementary material for: Differentiating care for persons with mild intellectual disability or borderline intellectual functioning: a Delphi study on the opinions of primary and professional caregivers and scientists
Source: BMC Psychiatry. 2020 Feb 10;20:57. doi: 10.1186/s12888-020-2437-4 (PMC7008567; doi:10.1186/s12888-020-2437-4)
Supplement: Supplementary file 2 — Additional file 2. Questionnaire: Round 2 Delphi study. [file 12888_2020_2437_MOESM2_ESM.docx]

**Questionnaire: Round 2 Delphi study**

**Introduction**

The goal of this Delphi study is to investigate what kind of support parents of individuals with mild intellectual disability (MID) or borderline intellectual function (BIF), researchers and professional caregivers would endorse per profile. The research consists of three rounds. Round 1 has been completed. Based on the answers from Round 1, we have formulated statements about the care for people with MID of BIF.

These statements are presented in this questionnaire. The questionnaire starts with a description of 1 of the 5 profiles; all the profiles are based on scientific research. Each description is followed by statements about the care and support for this particular profile. We would like to ask you to indicate to what extent you agree or disagree with these statements. Please note, there are no right or wrong answers, we are only interested in your own insights and ideas. Completing the questionnaire will probably take about 30 minutes.

Thank you in advance for your cooperation!

With kind regards,

Peter Nouwens, Nienke Smulders, Petri Embregts and Chijs van Nieuwenhuizen

**Profile description**

**Profile 1: Persons with mild intellectual disability**

The population included in this profile mainly consists of men with an average age of 27 years. Most of them have a mild intellectual disability; sometimes, they have a mild form of autism. Almost all persons in profile 1 have a day activity or work. However, because they have difficulty in maintaining social contacts, they have few friends. Their parents are competent in parenting and, in most cases, provide social/emotional support.

**Statements for persons in this profile:**

For persons in this profile:

1. The person’s socio-emotional development must be stimulated.

| Strongly disagree (1) | Disagree (2) | Neutral (3) | Agree (4) | Strongly agree (5) |
| --- | --- | --- | --- | --- |
|  |  |  |  |  |

1. Support is needed to enter into and maintain social contacts.

| Strongly disagree (1) | Disagree (2) | Neutral (3) | Agree (4) | Strongly agree (5) |
| --- | --- | --- | --- | --- |
|  |  |  |  |  |

1. Support from the individual’s own network (family, friends, acquaintances) is important.

| Strongly disagree (1) | Disagree (2) | Neutral (3) | Agree (4) | Strongly agree (5) |
| --- | --- | --- | --- | --- |
|  |  |  |  |  |

1. Requests for help must be examined together with the individual and the network.

| Strongly disagree (1) | Disagree (2) | Neutral (3) | Agree (4) | Strongly agree (5) |
| --- | --- | --- | --- | --- |
|  |  |  |  |  |

1. Attention to a person’s resilience level is important.

| Strongly disagree (1) | Disagree (2) | Neutral (3) | Agree (4) | Strongly agree (5) |
| --- | --- | --- | --- | --- |
|  |  |  |  |  |

1. Everyone should be approached as a ‘human being’, rather than as a ‘patient’.

| Strongly disagree (1) | Disagree (2) | Neutral (3) | Agree (4) | Strongly agree (5) |
| --- | --- | --- | --- | --- |
|  |  |  |  |  |

1. An explanation should be given about the impact of the disability on daily life.

| Strongly disagree (1) | Disagree (2) | Neutral (3) | Agree (4) | Strongly agree (5) |
| --- | --- | --- | --- | --- |
|  |  |  |  |  |

1. Support regarding fulfilling leisure time is essential.

| Strongly disagree (1) | Disagree (2) | Neutral (3) | Agree (4) | Strongly agree (5) |
| --- | --- | --- | --- | --- |
|  |  |  |  |  |

1. Guidance in independent living is indispensable.

| Strongly disagree (1) | Disagree (2) | Neutral (3) | Agree (4) | Strongly agree (5) |
| --- | --- | --- | --- | --- |
|  |  |  |  |  |

1. Support in finding and keeping daytime activities/work is very important.

| Strongly disagree (1) | Disagree (2) | Neutral (3) | Agree (4) | Strongly agree (5) |
| --- | --- | --- | --- | --- |
|  |  |  |  |  |

1. Guidance in finding adequate support is very important.

| Strongly disagree (1) | Disagree (2) | Neutral (3) | Agree (4) | Strongly agree (5) |
| --- | --- | --- | --- | --- |
|  |  |  |  |  |

1. Focus on an independent life is essential.

| Strongly disagree (1) | Disagree (2) | Neutral (3) | Agree (4) | Strongly agree (5) |
| --- | --- | --- | --- | --- |
|  |  |  |  |  |

1. Support from the person’s own network is necessary.

| Strongly disagree (1) | Disagree (2) | Neutral (3) | Agree (4) | Strongly agree (5) |
| --- | --- | --- | --- | --- |
|  |  |  |  |  |

1. Support for the general activities of daily living has priority.

| Strongly disagree (1) | Disagree (2) | Neutral (3) | Agree (4) | Strongly agree (5) |
| --- | --- | --- | --- | --- |
|  |  |  |  |  |

1. Guidance on finances is necessary.

| Strongly disagree (1) | Disagree (2) | Neutral (3) | Agree (4) | Strongly agree (5) |
| --- | --- | --- | --- | --- |
|  |  |  |  |  |

1. Training in social skills is essential.

| Strongly disagree (1) | Disagree (2) | Neutral (3) | Agree (4) | Strongly agree (5) |
| --- | --- | --- | --- | --- |
|  |  |  |  |  |

1. No support is needed.

| Strongly disagree (1) | Disagree (2) | Neutral (3) | Agree (4) | Strongly agree (5) |
| --- | --- | --- | --- | --- |
|  |  |  |  |  |

1. Knowledge on all aspects of the person (e.g. intellectual ability, social skills, participation in society) is necessary to achieve an optimal treatment and support program.

| Strongly disagree (1) | Disagree (2) | Neutral (3) | Agree (4) | Strongly agree (5) |
| --- | --- | --- | --- | --- |
|  |  |  |  |  |

1. The intensity of both treatment and support should easily be adjusted when necessary (a lot when required, a little when things are going well).

| Strongly disagree (1) | Disagree (2) | Neutral (3) | Agree (4) | Strongly agree (5) |
| --- | --- | --- | --- | --- |
|  |  |  |  |  |

1. Treatment and support must focus on the possibilities/strengths of the individual.

| Strongly disagree (1) | Disagree (2) | Neutral (3) | Agree (4) | Strongly agree (5) |
| --- | --- | --- | --- | --- |
|  |  |  |  |  |

1. Respite care is required to relieve the individual’s own network.

| Strongly disagree (1) | Disagree (2) | Neutral (3) | Agree (4) | Strongly agree (5) |
| --- | --- | --- | --- | --- |
|  |  |  |  |  |

1. The treatment and support offered should not feel like a ‘must’ or be forced.

| Strongly disagree (1) | Disagree (2) | Neutral (3) | Agree (4) | Strongly agree (5) |
| --- | --- | --- | --- | --- |
|  |  |  |  |  |

1. Both treatment and support should focus on participating in society.

| Strongly disagree (1) | Disagree (2) | Neutral (3) | Agree (4) | Strongly agree (5) |
| --- | --- | --- | --- | --- |
|  |  |  |  |  |

1. Individuals should have a personal budget which can be used for treatment, or to support themselves.

| Strongly disagree (1) | Disagree (2) | Neutral (3) | Agree (4) | Strongly agree (5) |
| --- | --- | --- | --- | --- |
|  |  |  |  |  |

1. Knowledge about the characteristics of persons in this profile is essential.

| Strongly disagree (1) | Disagree (2) | Neutral (3) | Agree (4) | Strongly agree (5) |
| --- | --- | --- | --- | --- |
|  |  |  |  |  |

1. Good alignment between different healthcare providers is essential.

| Strongly disagree (1) | Disagree (2) | Neutral (3) | Agree (4) | Strongly agree (5) |
| --- | --- | --- | --- | --- |
|  |  |  |  |  |

1. There is a lack of knowledge about the characteristics of these individuals in our society.

| Strongly disagree (1) | Disagree (2) | Neutral (3) | Agree (4) | Strongly agree (5) |
| --- | --- | --- | --- | --- |
|  |  |  |  |  |

**Profile description**

**Profile 2: Males with problem behaviour**

This profile consists mainly of men with borderline intellectual functioning and with behavioural problems; their average age is 25 years. Individuals in this profile have often been addicted to alcohol and/or drugs. Most of these persons experience difficulty in maintaining friendships. In the past, they may have had contact with the police and/or judicial authorities. Most have some form of day activity or work. Their parents are generally emotionally supportive, but had difficulty raising their child. Parents received almost no help in raising their child from family or friends. Some mothers of individuals in this profile have their own mental health problems.

**Statements for persons in this profile:**

For persons in this profile:

1. Appropriate research on additional problems, besides the intellectual disability, is required.

| Strongly disagree (1) | Disagree (2) | Neutral (3) | Agree (4) | Strongly agree (5) |
| --- | --- | --- | --- | --- |
|  |  |  |  |  |

1. An explanation should be given about the impact of the disability on daily life.

| Strongly disagree (1) | Disagree (2) | Neutral (3) | Agree (4) | Strongly agree (5) |
| --- | --- | --- | --- | --- |
|  |  |  |  |  |

1. A perspective for the future must be offered.

| Strongly disagree (1) | Disagree (2) | Neutral (3) | Agree (4) | Strongly agree (5) |
| --- | --- | --- | --- | --- |
|  |  |  |  |  |

1. Offering safety is a priority.

| Strongly disagree (1) | Disagree (2) | Neutral (3) | Agree (4) | Strongly agree (5) |
| --- | --- | --- | --- | --- |
|  |  |  |  |  |

1. Contact with persons in a similar situation is important.

| Strongly disagree (1) | Disagree (2) | Neutral (3) | Agree (4) | Strongly agree (5) |
| --- | --- | --- | --- | --- |
|  |  |  |  |  |

1. Clear boundaries and agreements are important.

| Strongly disagree (1) | Disagree (2) | Neutral (3) | Agree (4) | Strongly agree (5) |
| --- | --- | --- | --- | --- |
|  |  |  |  |  |

1. Treatment and support must focus on the possibilities/strengths of the individual.

| Strongly disagree (1) | Disagree (2) | Neutral (3) | Agree (4) | Strongly agree (5) |
| --- | --- | --- | --- | --- |
|  |  |  |  |  |

1. Earlier interventions are necessary (e.g. prior to the emergence of prominent problems).

| Strongly disagree (1) | Disagree (2) | Neutral (3) | Agree (4) | Strongly agree (5) |
| --- | --- | --- | --- | --- |
|  |  |  |  |  |

1. Support from the individual’s own network is important.

| Strongly disagree (1) | Disagree (2) | Neutral (3) | Agree (4) | Strongly agree (5) |
| --- | --- | --- | --- | --- |
|  |  |  |  |  |

1. Assistance in building and maintaining friendships is important.

| Strongly disagree (1) | Disagree (2) | Neutral (3) | Agree (4) | Strongly agree (5) |
| --- | --- | --- | --- | --- |
|  |  |  |  |  |

1. Support in finding and keeping daytime activities/work is very important.

| Strongly disagree (1) | Disagree (2) | Neutral (3) | Agree (4) | Strongly agree (5) |
| --- | --- | --- | --- | --- |
|  |  |  |  |  |

1. Guidance in the development towards independence is necessary.

| Strongly disagree (1) | Disagree (2) | Neutral (3) | Agree (4) | Strongly agree (5) |
| --- | --- | --- | --- | --- |
|  |  |  |  |  |

1. Support from the individual’s own network is inevitable.

| Strongly disagree (1) | Disagree (2) | Neutral (3) | Agree (4) | Strongly agree (5) |
| --- | --- | --- | --- | --- |
|  |  |  |  |  |

1. Guidance focused on participation in society is indispensable.

| Strongly disagree (1) | Disagree (2) | Neutral (3) | Agree (4) | Strongly agree (5) |
| --- | --- | --- | --- | --- |
|  |  |  |  |  |

1. Guidance on finances is necessary.

| Strongly disagree (1) | Disagree (2) | Neutral (3) | Agree (4) | Strongly agree (5) |
| --- | --- | --- | --- | --- |
|  |  |  |  |  |

1. A good relation between the individual and professional caregiver is essential.

| Strongly disagree (1) | Disagree (2) | Neutral (3) | Agree (4) | Strongly agree (5) |
| --- | --- | --- | --- | --- |
|  |  |  |  |  |

1. Accepting support should be stimulated.

| Strongly disagree (1) | Disagree (2) | Neutral (3) | Agree (4) | Strongly agree (5) |
| --- | --- | --- | --- | --- |
|  |  |  |  |  |

1. Training in social skills is indispensable.

| Strongly disagree (1) | Disagree (2) | Neutral (3) | Agree (4) | Strongly agree (5) |
| --- | --- | --- | --- | --- |
|  |  |  |  |  |

1. Resistance training should be offered.

| Strongly disagree (1) | Disagree (2) | Neutral (3) | Agree (4) | Strongly agree (5) |
| --- | --- | --- | --- | --- |
|  |  |  |  |  |

1. Cognitive behavioural therapy is required.

| Strongly disagree (1) | Disagree (2) | Neutral (3) | Agree (4) | Strongly agree (5) |
| --- | --- | --- | --- | --- |
|  |  |  |  |  |

1. Only evidence-based treatment should be offered.

| Strongly disagree (1) | Disagree (2) | Neutral (3) | Agree (4) | Strongly agree (5) |
| --- | --- | --- | --- | --- |
|  |  |  |  |  |

1. Treatment of behavioural problems is essential.

| Strongly disagree (1) | Disagree (2) | Neutral (3) | Agree (4) | Strongly agree (5) |
| --- | --- | --- | --- | --- |
|  |  |  |  |  |

1. Obligatory treatment is required.

| Strongly disagree (1) | Disagree (2) | Neutral (3) | Agree (4) | Strongly agree (5) |
| --- | --- | --- | --- | --- |
|  |  |  |  |  |

1. Treatment of mental health problems (if present) has priority.

| Strongly disagree (1) | Disagree (2) | Neutral (3) | Agree (4) | Strongly agree (5) |
| --- | --- | --- | --- | --- |
|  |  |  |  |  |

1. Treatment of an addiction (if present) has priority.

| Strongly disagree (1) | Disagree (2) | Neutral (3) | Agree (4) | Strongly agree (5) |
| --- | --- | --- | --- | --- |
|  |  |  |  |  |

1. A relapse in addiction must be prevented.

| Strongly disagree (1) | Disagree (2) | Neutral (3) | Agree (4) | Strongly agree (5) |
| --- | --- | --- | --- | --- |
|  |  |  |  |  |

1. The intellectual disability must be taken into account in the treatment of addiction.

| Strongly disagree (1) | Disagree (2) | Neutral (3) | Agree (4) | Strongly agree (5) |
| --- | --- | --- | --- | --- |
|  |  |  |  |  |

1. Semi-mural living with professional care is indispensable.

| Strongly disagree (1) | Disagree (2) | Neutral (3) | Agree (4) | Strongly agree (5) |
| --- | --- | --- | --- | --- |
|  |  |  |  |  |

1. Treatment by a multidisciplinary ambulant team (FACT) is required.

| Strongly disagree (1) | Disagree (2) | Neutral (3) | Agree (4) | Strongly agree (5) |
| --- | --- | --- | --- | --- |
|  |  |  |  |  |

1. Case management is necessary in which support and treatment are aligned.

| Strongly disagree (1) | Disagree (2) | Neutral (3) | Agree (4) | Strongly agree (5) |
| --- | --- | --- | --- | --- |
|  |  |  |  |  |

1. The intensity of treatment and support must be easily adjusted (a lot when necessary, a little when things are going well)

| Strongly disagree (1) | Disagree (2) | Neutral (3) | Agree (4) | Strongly agree (5) |
| --- | --- | --- | --- | --- |
|  |  |  |  |  |

1. Aftercare after cessation of treatment is indispensable.

| Strongly disagree (1) | Disagree (2) | Neutral (3) | Agree (4) | Strongly agree (5) |
| --- | --- | --- | --- | --- |
|  |  |  |  |  |

1. Ongoing examination of the personal requests and goals during treatment and support is important.

| Strongly disagree (1) | Disagree (2) | Neutral (3) | Agree (4) | Strongly agree (5) |
| --- | --- | --- | --- | --- |
|  |  |  |  |  |

1. Tenacity is required in seeking contact and offering support.

| Strongly disagree (1) | Disagree (2) | Neutral (3) | Agree (4) | Strongly agree (5) |
| --- | --- | --- | --- | --- |
|  |  |  |  |  |

1. Coordination of the various sectors in healthcare is important.

| Strongly disagree (1) | Disagree (2) | Neutral (3) | Agree (4) | Strongly agree (5) |
| --- | --- | --- | --- | --- |
|  |  |  |  |  |

1. Support in the care for persons with an intellectual disability is indispensable.

| Strongly disagree (1) | Disagree (2) | Neutral (3) | Agree (4) | Strongly agree (5) |
| --- | --- | --- | --- | --- |
|  |  |  |  |  |

1. Government must formulate specific policies.

| Strongly disagree (1) | Disagree (2) | Neutral (3) | Agree (4) | Strongly agree (5) |
| --- | --- | --- | --- | --- |
|  |  |  |  |  |

**Profile description**

**Profile 3: Persons with material hardship and abuse by parents**

Most persons in this profile are women with borderline intellectual functioning; their average age is 30 years. Some of the persons in this profile may have a mood disorder; furthermore, they often have debts. Most people in this profile have difficulties in maintaining friendships. A relatively large proportion of this group have been subjected to sexual and/or physical abuse by their parents; moreover, their parents were inconsistent in their upbringing style. The brothers and sisters of these individuals often have psychological problems themselves.

**Statements for persons in this profile:**

For persons in this profile:

1. Assistance in building and maintaining friendships is important.

| Strongly disagree (1) | Disagree (2) | Neutral (3) | Agree (4) | Strongly agree (5) |
| --- | --- | --- | --- | --- |
|  |  |  |  |  |

1. Support from the individual’s own network is important.

| Strongly disagree (1) | Disagree (2) | Neutral (3) | Agree (4) | Strongly agree (5) |
| --- | --- | --- | --- | --- |
|  |  |  |  |  |

1. The provision of a consistent and regular structure is necessary.

| Strongly disagree (1) | Disagree (2) | Neutral (3) | Agree (4) | Strongly agree (5) |
| --- | --- | --- | --- | --- |
|  |  |  |  |  |

1. An explanation should be given about the impact of the disability on daily life.

| Strongly disagree (1) | Disagree (2) | Neutral (3) | Agree (4) | Strongly agree (5) |
| --- | --- | --- | --- | --- |
|  |  |  |  |  |

1. It is essential that attention is paid to the qualities of the person.

| Strongly disagree (1) | Disagree (2) | Neutral (3) | Agree (4) | Strongly agree (5) |
| --- | --- | --- | --- | --- |
|  |  |  |  |  |

1. Stimulation of participation in society is important.

| Strongly disagree (1) | Disagree (2) | Neutral (3) | Agree (4) | Strongly agree (5) |
| --- | --- | --- | --- | --- |
|  |  |  |  |  |

1. Tenacity is required in seeking and maintaining contacts.

| Strongly disagree (1) | Disagree (2) | Neutral (3) | Agree (4) | Strongly agree (5) |
| --- | --- | --- | --- | --- |
|  |  |  |  |  |

1. Endurance is required in treatment and support.

| Strongly disagree (1) | Disagree (2) | Neutral (3) | Agree (4) | Strongly agree (5) |
| --- | --- | --- | --- | --- |
|  |  |  |  |  |

1. Support in developing self-confidence and a positive self-image is necessary.

| Strongly disagree (1) | Disagree (2) | Neutral (3) | Agree (4) | Strongly agree (5) |
| --- | --- | --- | --- | --- |
|  |  |  |  |  |

1. Support in finding and keeping daytime activities/work is very important.

| Strongly disagree (1) | Disagree (2) | Neutral (3) | Agree (4) | Strongly agree (5) |
| --- | --- | --- | --- | --- |
|  |  |  |  |  |

1. Support from the person’s own network is required.

| Strongly disagree (1) | Disagree (2) | Neutral (3) | Agree (4) | Strongly agree (5) |
| --- | --- | --- | --- | --- |
|  |  |  |  |  |

1. Guidance on finances and debts is necessary.

| Strongly disagree (1) | Disagree (2) | Neutral (3) | Agree (4) | Strongly agree (5) |
| --- | --- | --- | --- | --- |
|  |  |  |  |  |

1. Outpatient support in independent living is indispensable.

| Strongly disagree (1) | Disagree (2) | Neutral (3) | Agree (4) | Strongly agree (5) |
| --- | --- | --- | --- | --- |
|  |  |  |  |  |

1. Guidance on independency is necessary.

| Strongly disagree (1) | Disagree (2) | Neutral (3) | Agree (4) | Strongly agree (5) |
| --- | --- | --- | --- | --- |
|  |  |  |  |  |

1. Assistance is required in finding an appropriate education.

| Strongly disagree (1) | Disagree (2) | Neutral (3) | Agree (4) | Strongly agree (5) |
| --- | --- | --- | --- | --- |
|  |  |  |  |  |

1. Training in social skills is essential.

| Strongly disagree (1) | Disagree (2) | Neutral (3) | Agree (4) | Strongly agree (5) |
| --- | --- | --- | --- | --- |
|  |  |  |  |  |

1. Knowledge on all relevant aspects is necessary to achieve an optimal treatment and support program.

| Strongly disagree (1) | Disagree (2) | Neutral (3) | Agree (4) | Strongly agree (5) |
| --- | --- | --- | --- | --- |
|  |  |  |  |  |

1. Different forms of treatment are required to eliminate the causes of problems experienced by individuals.

| Strongly disagree (1) | Disagree (2) | Neutral (3) | Agree (4) | Strongly agree (5) |
| --- | --- | --- | --- | --- |
|  |  |  |  |  |

1. Creative professional therapy is necessary.

| Strongly disagree (1) | Disagree (2) | Neutral (3) | Agree (4) | Strongly agree (5) |
| --- | --- | --- | --- | --- |
|  |  |  |  |  |

1. Psychotherapy is required.

| Strongly disagree (1) | Disagree (2) | Neutral (3) | Agree (4) | Strongly agree (5) |
| --- | --- | --- | --- | --- |
|  |  |  |  |  |

1. Cognitive behavioural therapy is essential.

| Strongly disagree (1) | Disagree (2) | Neutral (3) | Agree (4) | Strongly agree (5) |
| --- | --- | --- | --- | --- |
|  |  |  |  |  |

1. Treatment must primarily focus on providing safety.

| Strongly disagree (1) | Disagree (2) | Neutral (3) | Agree (4) | Strongly agree (5) |
| --- | --- | --- | --- | --- |
|  |  |  |  |  |

1. Treatment by a multidisciplinary ambulant team (FACT) is required.

| Strongly disagree (1) | Disagree (2) | Neutral (3) | Agree (4) | Strongly agree (5) |
| --- | --- | --- | --- | --- |
|  |  |  |  |  |

1. Treatment in processing trauma is essential.

| Strongly disagree (1) | Disagree (2) | Neutral (3) | Agree (4) | Strongly agree (5) |
| --- | --- | --- | --- | --- |
|  |  |  |  |  |

1. Treatment of the mood disorder is required.

| Strongly disagree (1) | Disagree (2) | Neutral (3) | Agree (4) | Strongly agree (5) |
| --- | --- | --- | --- | --- |
|  |  |  |  |  |

1. Treatment of attachment problems is necessary.

| Strongly disagree (1) | Disagree (2) | Neutral (3) | Agree (4) | Strongly agree (5) |
| --- | --- | --- | --- | --- |
|  |  |  |  |  |

1. Obligatory treatment is necessary.

| Strongly disagree (1) | Disagree (2) | Neutral (3) | Agree (4) | Strongly agree (5) |
| --- | --- | --- | --- | --- |
|  |  |  |  |  |

1. Mental problems, if present, should have priority.

| Strongly disagree (1) | Disagree (2) | Neutral (3) | Agree (4) | Strongly agree (5) |
| --- | --- | --- | --- | --- |
|  |  |  |  |  |

1. Long-term support or treatment is required.

| Strongly disagree (1) | Disagree (2) | Neutral (3) | Agree (4) | Strongly agree (5) |
| --- | --- | --- | --- | --- |
|  |  |  |  |  |

1. Protection against persons in the environment related to abuse or maltreatment is important.

| Strongly disagree (1) | Disagree (2) | Neutral (3) | Agree (4) | Strongly agree (5) |
| --- | --- | --- | --- | --- |
|  |  |  |  |  |

1. The intensity of treatment and support should be easily adjusted (a lot when necessary, a little when things are going well).

| Strongly disagree (1) | Disagree (2) | Neutral (3) | Agree (4) | Strongly agree (5) |
| --- | --- | --- | --- | --- |
|  |  |  |  |  |

1. Early support is needed to prevent escalation of problems.

| Strongly disagree (1) | Disagree (2) | Neutral (3) | Agree (4) | Strongly agree (5) |
| --- | --- | --- | --- | --- |
|  |  |  |  |  |

1. An active and outreaching support approach of professional caregivers is necessary.

| Strongly disagree (1) | Disagree (2) | Neutral (3) | Agree (4) | Strongly agree (5) |
| --- | --- | --- | --- | --- |
|  |  |  |  |  |

1. A permanent core of professionals with few changes is required.

| Strongly disagree (1) | Disagree (2) | Neutral (3) | Agree (4) | Strongly agree (5) |
| --- | --- | --- | --- | --- |
|  |  |  |  |  |

1. Alignment between the different healthcare providers (involved with the family) is necessary.

| Strongly disagree (1) | Disagree (2) | Neutral (3) | Agree (4) | Strongly agree (5) |
| --- | --- | --- | --- | --- |
|  |  |  |  |  |

1. More knowledge is needed within the mental health sector.

| Strongly disagree (1) | Disagree (2) | Neutral (3) | Agree (4) | Strongly agree (5) |
| --- | --- | --- | --- | --- |
|  |  |  |  |  |

1. More knowledge/information is needed in our society.

| Strongly disagree (1) | Disagree (2) | Neutral (3) | Agree (4) | Strongly agree (5) |
| --- | --- | --- | --- | --- |
|  |  |  |  |  |

**Profile description**

**Profile 4: Male youngsters with problem behaviour and family problems**

This profile mainly consists of young men with borderline intellectual functioning; in this profile, persons with mild intellectual disability or borderline intellectual functioning have an average age of 19 years. All persons in this profile show behavioural problems. A relatively large proportion has been in contact with the police/judicial authorities, or has been in prison. All persons in this profile go to school. Although most of them have friends, they are surrounded by a vulnerable family system. All their parents are divorced and often also have financial problems; however, these parents receive a relatively large amount of informal support that might help their parenting.

**Statements for persons in this profile:**

For persons in this profile:

1. Creating a positive social network (friends) is important.

| Strongly disagree (1) | Disagree (2) | Neutral (3) | Agree (4) | Strongly agree (5) |
| --- | --- | --- | --- | --- |
|  |  |  |  |  |

1. Attention to a safe (home) environment is required.

| Strongly disagree (1) | Disagree (2) | Neutral (3) | Agree (4) | Strongly agree (5) |
| --- | --- | --- | --- | --- |
|  |  |  |  |  |

1. A safety net with appropriate persons in case of problems or relapse is important.

| Strongly disagree (1) | Disagree (2) | Neutral (3) | Agree (4) | Strongly agree (5) |
| --- | --- | --- | --- | --- |
|  |  |  |  |  |

1. A perspective for the future must be offered.

| Strongly disagree (1) | Disagree (2) | Neutral (3) | Agree (4) | Strongly agree (5) |
| --- | --- | --- | --- | --- |
|  |  |  |  |  |

1. Socio-emotional development must be stimulated.

| Strongly disagree (1) | Disagree (2) | Neutral (3) | Agree (4) | Strongly agree (5) |
| --- | --- | --- | --- | --- |
|  |  |  |  |  |

1. Appropriate work is essential.

| Strongly disagree (1) | Disagree (2) | Neutral (3) | Agree (4) | Strongly agree (5) |
| --- | --- | --- | --- | --- |
|  |  |  |  |  |

1. Extensive assessment of additional problems, besides the intellectual disability, is necessary.

| Strongly disagree (1) | Disagree (2) | Neutral (3) | Agree (4) | Strongly agree (5) |
| --- | --- | --- | --- | --- |
|  |  |  |  |  |

1. Careful identification of the potential risks a person is exposed to is important.

| Strongly disagree (1) | Disagree (2) | Neutral (3) | Agree (4) | Strongly agree (5) |
| --- | --- | --- | --- | --- |
|  |  |  |  |  |

1. Support should be aligned with the individual’s strengths.

| Strongly disagree (1) | Disagree (2) | Neutral (3) | Agree (4) | Strongly agree (5) |
| --- | --- | --- | --- | --- |
|  |  |  |  |  |

1. Support from the family is essential.

| Strongly disagree (1) | Disagree (2) | Neutral (3) | Agree (4) | Strongly agree (5) |
| --- | --- | --- | --- | --- |
|  |  |  |  |  |

1. Support in living independently is essential.

| Strongly disagree (1) | Disagree (2) | Neutral (3) | Agree (4) | Strongly agree (5) |
| --- | --- | --- | --- | --- |
|  |  |  |  |  |

1. Support regarding the existing group of friends is important.

| Strongly disagree (1) | Disagree (2) | Neutral (3) | Agree (4) | Strongly agree (5) |
| --- | --- | --- | --- | --- |
|  |  |  |  |  |

1. Support in education is needed.

| Strongly disagree (1) | Disagree (2) | Neutral (3) | Agree (4) | Strongly agree (5) |
| --- | --- | --- | --- | --- |
|  |  |  |  |  |

1. Support in the development of independency is essential.

| Strongly disagree (1) | Disagree (2) | Neutral (3) | Agree (4) | Strongly agree (5) |
| --- | --- | --- | --- | --- |
|  |  |  |  |  |

1. Guidance on participation in society is necessary.

| Strongly disagree (1) | Disagree (2) | Neutral (3) | Agree (4) | Strongly agree (5) |
| --- | --- | --- | --- | --- |
|  |  |  |  |  |

1. Guidance on finances is necessary.

| Strongly disagree (1) | Disagree (2) | Neutral (3) | Agree (4) | Strongly agree (5) |
| --- | --- | --- | --- | --- |
|  |  |  |  |  |

1. Support in general activities of daily living should have priority.

| Strongly disagree (1) | Disagree (2) | Neutral (3) | Agree (4) | Strongly agree (5) |
| --- | --- | --- | --- | --- |
|  |  |  |  |  |

1. Attention must be paid to the individual’s resilience and self-esteem.

| Strongly disagree (1) | Disagree (2) | Neutral (3) | Agree (4) | Strongly agree (5) |
| --- | --- | --- | --- | --- |
|  |  |  |  |  |

1. Solid guidance with clear rules and structures is indispensable.

| Strongly disagree (1) | Disagree (2) | Neutral (3) | Agree (4) | Strongly agree (5) |
| --- | --- | --- | --- | --- |
|  |  |  |  |  |

1. Assistance must always be accessible and available.

| Strongly disagree (1) | Disagree (2) | Neutral (3) | Agree (4) | Strongly agree (5) |
| --- | --- | --- | --- | --- |
|  |  |  |  |  |

1. A positive relation between the professional and individual is most important.

| Strongly disagree (1) | Disagree (2) | Neutral (3) | Agree (4) | Strongly agree (5) |
| --- | --- | --- | --- | --- |
|  |  |  |  |  |

1. Mistakes can be made during the support process in order to learn.

| Strongly disagree (1) | Disagree (2) | Neutral (3) | Agree (4) | Strongly agree (5) |
| --- | --- | --- | --- | --- |
|  |  |  |  |  |

1. Persons sentenced to legal conviction (e.g. community service) after committing a criminal offence, should be supervised in the performance of that service.

| Strongly disagree (1) | Disagree (2) | Neutral (3) | Agree (4) | Strongly agree (5) |
| --- | --- | --- | --- | --- |
|  |  |  |  |  |

1. Cognitive behavioural therapy is required.

| Strongly disagree (1) | Disagree (2) | Neutral (3) | Agree (4) | Strongly agree (5) |
| --- | --- | --- | --- | --- |
|  |  |  |  |  |

1. Treatment focused on criminal behaviour is needed.

| Strongly disagree (1) | Disagree (2) | Neutral (3) | Agree (4) | Strongly agree (5) |
| --- | --- | --- | --- | --- |
|  |  |  |  |  |

1. Treatment by a multidisciplinary ambulant team (FACT) is required.

| Strongly disagree (1) | Disagree (2) | Neutral (3) | Agree (4) | Strongly agree (5) |
| --- | --- | --- | --- | --- |
|  |  |  |  |  |

1. Only evidence-based treatment should be offered.

| Strongly disagree (1) | Disagree (2) | Neutral (3) | Agree (4) | Strongly agree (5) |
| --- | --- | --- | --- | --- |
|  |  |  |  |  |

1. Obligatory treatment is necessary.

| Strongly disagree (1) | Disagree (2) | Neutral (3) | Agree (4) | Strongly agree (5) |
| --- | --- | --- | --- | --- |
|  |  |  |  |  |

1. Wrap-around care is required.

| Strongly disagree (1) | Disagree (2) | Neutral (3) | Agree (4) | Strongly agree (5) |
| --- | --- | --- | --- | --- |
|  |  |  |  |  |

1. Easily accessible street work is necessary.

| Strongly disagree (1) | Disagree (2) | Neutral (3) | Agree (4) | Strongly agree (5) |
| --- | --- | --- | --- | --- |
|  |  |  |  |  |

1. Different forms of treatment are required to eliminate the causes of problems experienced by individuals.

| Strongly disagree (1) | Disagree (2) | Neutral (3) | Agree (4) | Strongly agree (5) |
| --- | --- | --- | --- | --- |
|  |  |  |  |  |

1. Case management is necessary in which support and treatment are aligned.

| Strongly disagree (1) | Disagree (2) | Neutral (3) | Agree (4) | Strongly agree (5) |
| --- | --- | --- | --- | --- |
|  |  |  |  |  |

1. Early support and treatment (e.g. prior to the emergence of problems) is necessary.

| Strongly disagree (1) | Disagree (2) | Neutral (3) | Agree (4) | Strongly agree (5) |
| --- | --- | --- | --- | --- |
|  |  |  |  |  |

1. Treatment and support should remain the same when a person reaches 18 years of age.

| Strongly disagree (1) | Disagree (2) | Neutral (3) | Agree (4) | Strongly agree (5) |
| --- | --- | --- | --- | --- |
|  |  |  |  |  |

1. Cooperation between the different healthcare providers involved is necessary.

| Strongly disagree (1) | Disagree (2) | Neutral (3) | Agree (4) | Strongly agree (5) |
| --- | --- | --- | --- | --- |
|  |  |  |  |  |

**Profile description**

**Profile 5:** **Persons with addictive problems**

Most people in this profile have borderline intellectual functioning and behavioural problems, and all are addicted to alcohol and/or drugs. This subgroup profile has an almost equal distribution between men and women; the average age is 28 years. More than half of these persons have no permanent residence or home. No-one in this profile has any form of daytime activities or work. Also, most of them have difficulty establishing relationships with their peers. A relatively large proportion has contact with the criminal world and/or contact with the police and judicial authorities. Debts are common. Most persons in this profile have a partner, and some have one or more children. Generally, the family in which they were raised had various problems; most of their parents are divorced. Many persons in this group were abused by their parents. In addition, many of the parents had debts, mental health problems, and also had difficulty raising their children in a consistent and appropriate way.

**Statements for persons in this profile:**

For persons in this profile:

1. Housing is the first priority.

| Strongly disagree (1) | Disagree (2) | Neutral (3) | Agree (4) | Strongly agree (5) |
| --- | --- | --- | --- | --- |
|  |  |  |  |  |

1. Accepting support should be stimulated.

| Strongly disagree (1) | Disagree (2) | Neutral (3) | Agree (4) | Strongly agree (5) |
| --- | --- | --- | --- | --- |
|  |  |  |  |  |

1. Support in finding and keeping daytime activities/work is very important.

| Strongly disagree (1) | Disagree (2) | Neutral (3) | Agree (4) | Strongly agree (5) |
| --- | --- | --- | --- | --- |
|  |  |  |  |  |

1. A perspective for the future must be offered.

| Strongly disagree (1) | Disagree (2) | Neutral (3) | Agree (4) | Strongly agree (5) |
| --- | --- | --- | --- | --- |
|  |  |  |  |  |

1. A positive approach is essential.

| Strongly disagree (1) | Disagree (2) | Neutral (3) | Agree (4) | Strongly agree (5) |
| --- | --- | --- | --- | --- |
|  |  |  |  |  |

1. Realising a consistent and regular day structure is essential.

| Strongly disagree (1) | Disagree (2) | Neutral (3) | Agree (4) | Strongly agree (5) |
| --- | --- | --- | --- | --- |
|  |  |  |  |  |

1. Parenting support should be provided to ensure that the children are safely raised.

| Strongly disagree (1) | Disagree (2) | Neutral (3) | Agree (4) | Strongly agree (5) |
| --- | --- | --- | --- | --- |
|  |  |  |  |  |

1. Support regarding a safe (home) environment required.

| Strongly disagree (1) | Disagree (2) | Neutral (3) | Agree (4) | Strongly agree (5) |
| --- | --- | --- | --- | --- |
|  |  |  |  |  |

1. Participation in society should be encouraged.

| Strongly disagree (1) | Disagree (2) | Neutral (3) | Agree (4) | Strongly agree (5) |
| --- | --- | --- | --- | --- |
|  |  |  |  |  |

1. Attention for criminal behaviour is necessary.

| Strongly disagree (1) | Disagree (2) | Neutral (3) | Agree (4) | Strongly agree (5) |
| --- | --- | --- | --- | --- |
|  |  |  |  |  |

1. Support should focus on the basic necessities of life.

| Strongly disagree (1) | Disagree (2) | Neutral (3) | Agree (4) | Strongly agree (5) |
| --- | --- | --- | --- | --- |
|  |  |  |  |  |

1. Supporting the development of independence is necessary.

| Strongly disagree (1) | Disagree (2) | Neutral (3) | Agree (4) | Strongly agree (5) |
| --- | --- | --- | --- | --- |
|  |  |  |  |  |

1. Guidance in creating a supportive network is necessary.

| Strongly disagree (1) | Disagree (2) | Neutral (3) | Agree (4) | Strongly agree (5) |
| --- | --- | --- | --- | --- |
|  |  |  |  |  |

1. Support in developing self-confidence and a positive self-image is necessary.

| Strongly disagree (1) | Disagree (2) | Neutral (3) | Agree (4) | Strongly agree (5) |
| --- | --- | --- | --- | --- |
|  |  |  |  |  |

1. Guidance in clearing financial debts is a priority before support of the other issues.

| Strongly disagree (1) | Disagree (2) | Neutral (3) | Agree (4) | Strongly agree (5) |
| --- | --- | --- | --- | --- |
|  |  |  |  |  |

1. Support from a permanent ‘buddy’ is important.

| Strongly disagree (1) | Disagree (2) | Neutral (3) | Agree (4) | Strongly agree (5) |
| --- | --- | --- | --- | --- |
|  |  |  |  |  |

1. Permanent accessibility and availability of support is required.

| Strongly disagree (1) | Disagree (2) | Neutral (3) | Agree (4) | Strongly agree (5) |
| --- | --- | --- | --- | --- |
|  |  |  |  |  |

1. Long-term support is necessary.

| Strongly disagree (1) | Disagree (2) | Neutral (3) | Agree (4) | Strongly agree (5) |
| --- | --- | --- | --- | --- |
|  |  |  |  |  |

1. Early support is necessary to prevent escalation of problems.

| Strongly disagree (1) | Disagree (2) | Neutral (3) | Agree (4) | Strongly agree (5) |
| --- | --- | --- | --- | --- |
|  |  |  |  |  |

1. Support should include the whole system (family, parents, partner, and their own children).

| Strongly disagree (1) | Disagree (2) | Neutral (3) | Agree (4) | Strongly agree (5) |
| --- | --- | --- | --- | --- |
|  |  |  |  |  |

1. Treatment of the addiction should have priority.

| Strongly disagree (1) | Disagree (2) | Neutral (3) | Agree (4) | Strongly agree (5) |
| --- | --- | --- | --- | --- |
|  |  |  |  |  |

1. Treatment of problem behaviour is indispensable.

| Strongly disagree (1) | Disagree (2) | Neutral (3) | Agree (4) | Strongly agree (5) |
| --- | --- | --- | --- | --- |
|  |  |  |  |  |

1. Attention to possible attachment problems is essential.

| Strongly disagree (1) | Disagree (2) | Neutral (3) | Agree (4) | Strongly agree (5) |
| --- | --- | --- | --- | --- |
|  |  |  |  |  |

1. Obligatory support is necessary.

| Strongly disagree (1) | Disagree (2) | Neutral (3) | Agree (4) | Strongly agree (5) |
| --- | --- | --- | --- | --- |
|  |  |  |  |  |

1. Temporary in-patient care is inevitable.

| Strongly disagree (1) | Disagree (2) | Neutral (3) | Agree (4) | Strongly agree (5) |
| --- | --- | --- | --- | --- |
|  |  |  |  |  |

1. Treatment by a multidisciplinary ambulant team (FACT) is required.

| Strongly disagree (1) | Disagree (2) | Neutral (3) | Agree (4) | Strongly agree (5) |
| --- | --- | --- | --- | --- |
|  |  |  |  |  |

1. Case management is necessary in which support and treatment are aligned.

| Strongly disagree (1) | Disagree (2) | Neutral (3) | Agree (4) | Strongly agree (5) |
| --- | --- | --- | --- | --- |
|  |  |  |  |  |

1. Easily accessible street work is necessary.

| Strongly disagree (1) | Disagree (2) | Neutral (3) | Agree (4) | Strongly agree (5) |
| --- | --- | --- | --- | --- |
|  |  |  |  |  |

1. Wrap-around care is required.

| Strongly disagree (1) | Disagree (2) | Neutral (3) | Agree (4) | Strrongly agree (5) |
| --- | --- | --- | --- | --- |
|  |  |  |  |  |

1. Long-term interdisciplinary treatment is essential.

| Strongly disagree (1) | Disagree (2) | Neutral (3) | Agree (4) | Strongly agree (5) |
| --- | --- | --- | --- | --- |
|  |  |  |  |  |

1. Different forms of treatment are necessary to eliminate the causes of problems experienced by individuals.

| Strongly disagree (1) | Disagree (2) | Neutral (3) | Agree (4) | Strongly agree (5) |
| --- | --- | --- | --- | --- |
|  |  |  |  |  |

1. Tenacity and endurance are essential in treatment.

| Strongly disagree (1) | Disagree (2) | Neutral (3) | Agree (4) | Strongly agree (5) |
| --- | --- | --- | --- | --- |
|  |  |  |  |  |

1. The intellectual disability should be taken into account during treatment and support.

| Strongly disagree (1) | Disagree (2) | Neutral (3) | Agree (4) | Strongly agree (5) |
| --- | --- | --- | --- | --- |
|  |  |  |  |  |

1. It is important that professionals ‘free’ themselves from their own standards and values.

| Strongly disagree (1) | Disagree (2) | Neutral (3) | Agree (4) | Strongly agree (5) |
| --- | --- | --- | --- | --- |
|  |  |  |  |  |

1. Guidance on reintegration into society after completion of treatment and support is indispensable.

| Strongly disagree (1) | Disagree (2) | Neutral (3) | Agree (4) | Strongly agree (5) |
| --- | --- | --- | --- | --- |
|  |  |  |  |  |

1. Cooperation between different healthcare organisations is very important.

| Strongly disagree (1) | Disagree (2) | Neutral (3) | Agree (4) | Strongly agree (5) |
| --- | --- | --- | --- | --- |
|  |  |  |  |  |

1. One entrance to different forms of treatment and support is essential.

| Strongly disagree (1) | Disagree (2) | Neutral (3) | Agree (4) | Strongly agree (5) |
| --- | --- | --- | --- | --- |
|  |  |  |  |  |

1. More funds are required from government for treatment and support.

| Strongly disagree (1) | Disagree (2) | Neutral (3) | Agree (4) | Strongly agree (5) |
| --- | --- | --- | --- | --- |
|  |  |  |  |  |
