# Supplementary material for: Differentiating care for persons with mild intellectual disability or borderline intellectual functioning: a Delphi study on the opinions of primary and professional caregivers and scientists
Source: BMC Psychiatry. 2020 Feb 10;20:57. doi: 10.1186/s12888-020-2437-4 (PMC7008567; doi:10.1186/s12888-020-2437-4)
Supplement: Supplementary file 3 — Additional file 3. Questionnaire: Round 3 Delphi study. [file 12888_2020_2437_MOESM3_ESM.docx]

**Questionnaire: Round 3 Delphi study**

The goal of this Delphi study is to reach consensus between expert groups about appropriate care for persons with mild intellectual disability (MID) or borderline intellectual function (BIF).
Using this Delphi study, we aim to investigate what kind of support parents of individuals with MID or BIF, researchers and professional caregivers would endorse per profile.

This third and final round includes a questionnaire that is similar to Round 2. However, the questionnaire in Round 3 contains **67 statements upon which consensus can still be reached**.

The questionnaire starts with a description of 1 of the 5 profiles; all the profiles are based on scientific research. Each description is followed by statements about the care and support for this particular profile.

Per statement we inform you about the degree (percentages) to which scientists, professional caregivers and primary caregivers endorsed (‘Agree’ and ‘Strongly agree’) these statements in Round 2. For the percentages presented below, ‘Agree’ and ‘Strongly agree’ are pooled to **give one percentage** showing the level of endorsement for each statement.

We would like to ask you to again indicate to what extent you agree with these 67 statements.

Please note, there are no right or wrong answers, we are only interested in your own insights and ideas. Completing the questionnaire will probably take about 30 minutes.

Thank you in advance for your cooperation!

With kind regards,

Peter Nouwens, Nienke Smulders, Petri Embregts and Chijs van Nieuwenhuizen

**Profile description**

**Profile 1: Persons with mild intellectual disability**

The population included in this profile mainly consists of men with an average age of 27 years. Most of them have a mild intellectual disability; sometimes, they have a mild form of autism. Almost all persons in profile 1 have a day activity or work. However, because they have difficulty in maintaining social contacts, they have few friends. Their parents are competent in parenting and, in most cases, provide social/emotional support.

**Statements for persons in this profile:**We would like to ask you again to indicate to what extent you agree with these statements.
For each statement you will see the percentages (pooled ‘Agree’ and ‘Strongly agree’) per expert group that emerged from Round 2.

For persons in this profile:

1. The person’s socio-emotional development must be stimulated.

In Round 2: scientists **61.5%,** professional caregivers **66.7%,** primary caregivers **94.7%.**

| Strongly disagree (1) | Disagree (2) | Neutral (3) | Agree (4) | Strongly agree (5) |
| --- | --- | --- | --- | --- |
|  |  |  |  |  |

1. Support is needed to enter into and maintain social contacts.

In Round 2: scientists **69.2%,** professional caregivers **95.2%,** primary caregivers **100%**.

| Strongly disagree (1) | Disagree (2) | Neutral (3) | Agree (4) | Strongly agree (5) |
| --- | --- | --- | --- | --- |
|  |  |  |  |  |

1. Attention to a person’s resilience level is important.
   In Round 2: scientists **61.6%,** professional caregivers **90.5%,** primary caregivers **100%**.

| Strongly disagree (1) | Disagree (2) | Neutral (3) | Agree (4) | Strongly agree (5) |
| --- | --- | --- | --- | --- |
|  |  |  |  |  |

1. Guidance in independent living is indispensable.

In Round 2: scientists **46.2%,** professional caregivers **57.2%,** primary caregivers **89.4%.**

| Strongly disagree (1) | Disagree (2) | Neutral (3) | Agree (4) | Strongly agree (5) |
| --- | --- | --- | --- | --- |
|  |  |  |  |  |

1. Support in finding and keeping daytime activities/work is very important.

In Round 2: scientists **69.2%,** professional caregivers **90.4%,** primary caregivers **89.5%**.

| Strongly disagree (1) | Disagree (2) | Neutral (3) | Agree (4) | Strongly agree (5) |
| --- | --- | --- | --- | --- |
|  |  |  |  |  |

1. Guidance in finding adequate support is very important.

In Round 2: scientists **30.8%,** professional caregivers **71.4%,** primary caregivers **94.7%**.

| Strongly disagree (1) | Disagree (2) | Neutral (3) | Agree (4) | Strongly agree (5) |
| --- | --- | --- | --- | --- |
|  |  |  |  |  |

1. Focus on an independent life is essential.

In Round 2: scientists **77%,** professional caregivers **71.5%,** primary caregivers **84.2%.**

| Strongly disagree (1) | Disagree (2) | Neutral (3) | Agree (4) | Strongly agree (5) |
| --- | --- | --- | --- | --- |
|  |  |  |  |  |

1. Training in social skills is essential.

In Round 2: scientists **46.2%,** professional caregivers **42.9%,** primary caregivers **84.2%.**

| Strongly disagree (1) | Disagree (2) | Neutral (3) | Agree (4) | Strongly agree (5) |
| --- | --- | --- | --- | --- |
|  |  |  |  |  |

1. Knowledge about the characteristics of persons in this profile is essential.

In Round 2: scientists **69.3%,** professional caregivers **80.9%,** primary caregivers **94.7%.**

| Strongly disagree (1) | Disagree (2) | Neutral (3) | Agree (4) | Strongly agree (5) |
| --- | --- | --- | --- | --- |
|  |  |  |  |  |

**Profile description**

**Profile 2: Males with problem behaviour**

This profile consists mainly of men with borderline intellectual functioning and with behavioural problems; their average age is 25 years. Individuals in this profile have often been addicted to alcohol and/or drugs. Most of these persons experience difficulty in maintaining friendships. In the past, they may have had contact with the police and/or judicial authorities. Most have some form of day activity or work. Their parents are generally emotionally supportive, but had difficulty raising their child. Parents received almost no help in raising their child from family or friends. Some mothers of individuals in this profile have their own mental health problems.

**Statements for persons in this profile:**We would like to ask you again to indicate to what extent you agree with these statements.
For each statement you will see the percentages (pooled ‘Agree’ and ‘Strongly agree’) per expert group that emerged from Round 2.

For persons in this profile:

1. An explanation should be given about the impact of the disability on daily life.

In Round 2: scientists **69.3%,** professional caregivers **85.7%,** primary caregivers **89.5%.**

| Strongly disagree (1) | Disagree (2) | Neutral (3) | Agree (4) | Strongly agree (5) |
| --- | --- | --- | --- | --- |
|  |  |  |  |  |

1. A perspective for the future must be offered.

In Round 2: scientists **61.6%,** professional caregivers **80.9%,** primary caregivers **78.9%.**

| Strongly disagree (1) | Disagree (2) | Neutral (3) | Agree (4) | Strongly agree (5) |
| --- | --- | --- | --- | --- |
|  |  |  |  |  |

1. Offering safety is a priority.

In Round 2: scientists **53.9%,** professional caregivers **61.9%,** primary caregivers **89.4%.**

| Strongly disagree (1) | Disagree (2) | Neutral (3) | Agree (4) | Strongly agree (5) |
| --- | --- | --- | --- | --- |
|  |  |  |  |  |

1. Clear boundaries and agreements are important

In Round 2: scientists **76.9%,** professional caregivers **80.9%,** primary caregivers **100%.**

| Strongly disagree (1) | Disagree (2) | Neutral (3) | Agree (4) | Strongly agree (5) |
| --- | --- | --- | --- | --- |
|  |  |  |  |  |

1. Assistance in building and maintaining friendships is important.

In Round 2: scientists **46.2%,** professional caregivers **89.5%,** primary caregivers **89.5%.**

| Strongly disagree (1) | Disagree (2) | Neutral (3) | Agree (4) | Strongly agree (5) |
| --- | --- | --- | --- | --- |
|  |  |  |  |  |

1. Support in finding and keeping daytime activities/work is very important.

In Round 2: scientists **77%,** professional caregivers **90.5%,** primary caregivers **89.5%.**

| Strongly disagree (1) | Disagree (2) | Neutral (3) | Agree (4) | Strongly agree (5) |
| --- | --- | --- | --- | --- |
|  |  |  |  |  |

1. Guidance on finances is necessary.

In Round 2: scientists **53.9%,** professional caregivers **76.2%,** primary caregivers **89.5%.**

| Strongly disagree (1) | Disagree (2) | Neutral (3) | Agree (4) | Strongly agree (5) |
| --- | --- | --- | --- | --- |
|  |  |  |  |  |

1. Accepting support should be stimulated.

In Round 2: scientists **77%,** professional caregivers **80.9%,** primary caregivers **94.7%.**

| Strongly disagree (1) | Disagree (2) | Neutral (3) | Agree (4) | Strongly agree (5) |
| --- | --- | --- | --- | --- |
|  |  |  |  |  |

1. Treatment of behavioural problems is essential.

In Round 2: scientists **76.9%,** professional caregivers **42.9%,** primary caregivers **84.3%.**

| Strongly disagree (1) | Disagree (2) | Neutral (3) | Agree (4) | Strongly agree (5) |
| --- | --- | --- | --- | --- |
|  |  |  |  |  |

1. A relapse in addiction must be prevented.

In Round 2: scientists **84.6%,** professional caregivers **76.2%,** primary caregivers **100%.**

| Strongly disagree (1) | Disagree (2) | Neutral (3) | Agree (4) | Strongly agree (5) |
| --- | --- | --- | --- | --- |
|  |  |  |  |  |

1. Treatment by a multidisciplinary ambulant team (FACT) is required.

In Round 2: scientists **84.6%,** professional caregivers **85.7%,** primary caregivers **73.7%.**

| Strongly disagree (1) | Disagree (2) | Neutral (3) | Agree (4) | Strongly agree (5) |
| --- | --- | --- | --- | --- |
|  |  |  |  |  |

1. Tenacity is required in seeking contact and offering support.

In Round 2: scientists **69.3%,** professional caregivers **90.5%,** primary caregivers **94.7%.**

| Strongly disagree (1) | Disagree (2) | Neutral (3) | Agree (4) | Strongly agree (5) |
| --- | --- | --- | --- | --- |
|  |  |  |  |  |

**Profile description**

**Profile 3: Persons with material hardship and abuse by parents**

Most persons in this profile are women with borderline intellectual functioning; their average age is 30 years. Some of the persons in this profile may have a mood disorder; furthermore, they often have debts. Most people in this profile have difficulties in maintaining friendships. A relatively large proportion of this group have been subjected to sexual and/or physical abuse by their parents; moreover, their parents were inconsistent in their upbringing style. The brothers and sisters of these individuals often have psychological problems themselves.

**Statements for persons in this profile:**

We would like to ask you again to indicate to what extent you agree with these statements.
For each statement you will see the percentages (pooled ‘Agree’ and ‘Strongly agree’) per expert group that emerged from Round 2.

For persons in this profile:

1. Assistance in building and maintaining friendships is important.

In Round 2: scientists **69.3%,** professional caregivers **90.4%,** primary caregivers **84.2%.**

| Strongly disagree (1) | Disagree (2) | Neutral (3) | Agree (4) | Strongly agree (5) |
| --- | --- | --- | --- | --- |
|  |  |  |  |  |

1. The provision of a consistent and regular structure is necessary.

In Round 2: scientists **76.9%,** professional caregivers **85.7%,** primary caregivers **94.7%.**

| Strongly disagree (1) | Disagree (2) | Neutral (3) | Agree (4) | Strongly agree (5) |
| --- | --- | --- | --- | --- |
|  |  |  |  |  |

1. An explanation should be given about the impact of the disability on daily life.

In Round 2: scientists **76.9%,** professional caregivers **80.9%,** primary caregivers **94.7%.**

| Strongly disagree (1) | Disagree (2) | Neutral (3) | Agree (4) | Strongly agree (5) |
| --- | --- | --- | --- | --- |
|  |  |  |  |  |

1. Stimulation of participation in society is important.

In Round 2: scientists **77%,** professional caregivers **85.7%,** primary caregivers **89.5%.**.

| Strongly disagree (1) | Disagree (2) | Neutral (3) | Agree (4) | Strongly agree (5) |
| --- | --- | --- | --- | --- |
|  |  |  |  |  |

1. Endurance in treatment and support is required.

In Round 2: scientists **92.3%,** professional caregivers **90.5%,** primary caregivers **78.9%.**

| Strongly disagree (1) | Disagree (2) | Neutral (3) | Agree (4) | Strongly agree (5) |
| --- | --- | --- | --- | --- |
|  |  |  |  |  |

1. Support in finding and keeping daytime activities/is work very important.

In Round 2: scientists **69.3%,** professional caregivers **90.5%,** primary caregivers **94.7%.**

| Strongly disagree (1) | Disagree (2) | Neutral (3) | Agree (4) | Strongly agree (5) |
| --- | --- | --- | --- | --- |
|  |  |  |  |  |

1. Guidance on finances and debts is necessary.

In Round 2: scientists **69.3%,** professional caregivers **90.5%,** primary caregivers **94.7%**.

| Strongly disagree (1) | Disagree (2) | Neutral (3) | Agree (4) | Strongly agree (5) |
| --- | --- | --- | --- | --- |
|  |  |  |  |  |

1. Outpatient support in independent living is indispensable.

In Round 2: scientists **77%,** professional caregivers **71.4%,** primary caregivers **100%.**

| Strongly disagree (1) | Disagree (2) | Neutral (3) | Agree (4) | Strongly agree (5) |
| --- | --- | --- | --- | --- |
|  |  |  |  |  |

1. Guidance on independency is necessary.

In Round 2: scientists **69.2%,** professional caregivers **80.9%,** primary caregivers **89.5%**.

| strongly disagree (1) | Disagree (2) | Neutral (3) | Agree (4) | Strongly agree (5) |
| --- | --- | --- | --- | --- |
|  |  |  |  |  |

1. Training in social skills is essential.

In Round 2: scientists **38.5%,** professional caregivers **57.2%,** primary caregivers **94.7%**.

| Strongly disagree (1) | Disagree (2) | Neutral (3) | Agree (4) | Strongly agree (5) |
| --- | --- | --- | --- | --- |
|  |  |  |  |  |

1. Different forms of treatment are required to eliminate the causes of problems experienced by individuals.

In Round 2: scientists **61.6%,** professional caregivers **61.9%,** primary caregivers **89.5%**.

| Strongly disagree (1) | Disagree (2) | Neutral (3) | Agree (4) | Strongly agree (5) |
| --- | --- | --- | --- | --- |
|  |  |  |  |  |

1. Psychotherapy is required.

In Round 2: scientists **38.5%,** professional caregivers **14.3%,** primary caregivers **89.4%.**

| Strongly disagree (1) | Disagree (2) | Neutral (3) | Agree (4) | Strongly agree (5) |
| --- | --- | --- | --- | --- |
|  |  |  |  |  |

1. Treatment in processing trauma is essential.

In Round 2: scientists **69.3%,** professional caregivers **71.4%,** primary caregivers **84.2%.**

| Strongly disagree (1) | Disagree (2) | Neutral (3) | Agree (4) | Strongly agree (5) |
| --- | --- | --- | --- | --- |
|  |  |  |  |  |

1. Treatment of the mood disorder is essential.

In Round 2: scientists **76.9%,** professional caregivers **71.4%,** primary caregivers **73.7%**.

| Strongly disagree (1) | Disagree (2) | Neutral (3) | Agree (4) | Strongly agree (5) |
| --- | --- | --- | --- | --- |
|  |  |  |  |  |

1. Treatment of attachment problems is necessary.

In Round 2: scientists **76.9%,** professional caregivers **52.3%,** primary caregivers **84.2%.**

| Strongly disagree (1) | Disagree (2) | Neutral (3) | Agree (4) | Strongly agree (5) |
| --- | --- | --- | --- | --- |
|  |  |  |  |  |

1. Long-term support or treatment is required.

In Round 2: scientists **77%,** professional caregivers **85.7%,** primary caregivers **78.9%.**

| Strongly disagree (1) | Disagree (2) | Neutral (3) | Agree (4) | Strongly agree (5) |
| --- | --- | --- | --- | --- |
|  |  |  |  |  |

1. An active, outreaching support approach of professional caregivers is necessary.

In Round 2: scientists **46.2%,** professional caregivers **85.7%,** primary caregivers **79%**.

| Strongly disagree (1) | Disagree (2) | Neutral (3) | Agree (4) | Strongly agree (5) |
| --- | --- | --- | --- | --- |
|  |  |  |  |  |

1. More knowledge is needed within the mental health sector.

In Round 2: scientists **84.6%,** professional caregivers **90.5%,** primary caregivers **57.9%.**

| Strongly disagree (1) | Disagree (2) | Neutral (3) | Agree (4) | Strongly agree (5) |
| --- | --- | --- | --- | --- |
|  |  |  |  |  |

1. More knowledge in our society is necessary.

In Round 2: scientists **77%,** professional caregivers **80.9%,** primary caregivers **73.6%.**

| Strongly disagree (1) | Disagree (2) | Neutral (3) | Agree (4) | Strongly agree (5) |
| --- | --- | --- | --- | --- |
|  |  |  |  |  |

**Profile description**

**Profile 4: Male youngsters with problem behaviour and family problems**

This profile mainly consists of young men with borderline intellectual functioning; in this profile, persons with mild intellectual disability or borderline intellectual functioning have an average age of 19 years. All persons in this profile show behavioural problems. A relatively large proportion has been in contact with the police/judicial authorities, or has been in prison. All persons in this profile go to school. Although most of them have friends, they are surrounded by a vulnerable family system. All their parents are divorced and often also have financial problems; however, these parents receive a relatively large amount of informal support that might help their parenting.

**Statements for persons in this profile:**

We would like to ask you again to indicate to what extent you agree with these statements.
For each statement you will see the percentages (pooled ‘Agree’ and ‘Strongly agree’) per expert group that emerged from Round 2.

For persons in this profile:

1. A perspective for the future must be offered.

In Round 2: scientists **76.9%,** professional caregivers **100%,** primary caregivers **94.7%**.

| Strongly disagree (1) | Disagree (2) | Neutral (3) | Agree (4) | Strongly agree (5) |
| --- | --- | --- | --- | --- |
|  |  |  |  |  |

1. Socio-emotional development must be stimulated.

In Round 2: scientists **69.2%,** professional caregivers **81%,** primary caregivers **84.2%**.

| Strongly disagree (1) | Disagree (2) | Neutral (3) | Agree (4) | Strongly agree (5) |
| --- | --- | --- | --- | --- |
|  |  |  |  |  |

1. Extensive assessment of additional problems, besides the intellectual disability, is necessary.

In Round 2: scientists **77%,** professional caregivers **81.4%,** primary caregivers **84.2%**.

| Strongly disagree (1) | Disagree (2) | Neutral (3) | Agree (4) | Strongly agree (5) |
| --- | --- | --- | --- | --- |
|  |  |  |  |  |

1. Support from the individual’s family is essential.

In Round 2: scientists **77%,** professional caregivers **85.7%,** primary caregivers **68.4%**.

| Strongly disagree (1) | Disagree (2) | Neutral (3) | Agree (4) | Strongly agree (5) |
| --- | --- | --- | --- | --- |
|  |  |  |  |  |

1. Support with living independently is essential.

In Round 2: scientists **84.7%,** professional caregivers **76.2%,** primary caregivers **94.8%**.

| Strongly disagree (1) | Disagree (2) | Neutral (3) | Agree (4) | Strongly agree (5) |
| --- | --- | --- | --- | --- |
|  |  |  |  |  |

1. Support in education is required.

In Round 2: scientists **61.8%,** professional caregivers **76.2%,** primary caregivers **84.2%**.

| Strongly disagree (1) | Disagree (2) | Neutral (3) | Agree (4) | Strongly agree (5) |
| --- | --- | --- | --- | --- |
|  |  |  |  |  |

1. Support with regard to the development of independency is essential.

In Round 2: scientists **69.3%,** professional caregivers **80.9%,** primary caregivers **100%**.

| Strongly disagree (1) | Disagree (2) | Neutral (3) | Agree (4) | Strongly agree (5) |
| --- | --- | --- | --- | --- |
|  |  |  |  |  |

1. Guidance regarding participation in society is necessary.

In Round 2: scientists **53.9%,** professional caregivers **80.9%,** primary caregivers **94.8%**.

| Strongly disagree (1) | Disagree (2) | Neutral (3) | Agree (4) | Strongly agree (5) |
| --- | --- | --- | --- | --- |
|  |  |  |  |  |

1. Guidance on finances is necessary.

In Round 2: scientists **69.3%,** professional caregivers **85.7%,** primary caregivers **89.5%**.

| Strongly disagree (1) | Disagree (2) | Neutral (3) | Agree (4) | Strongly agree (5) |
| --- | --- | --- | --- | --- |
|  |  |  |  |  |

1. Attention for a person’s resilience and self-esteem is necessary.

In Round 2: scientists **69.3%,** professional caregivers **71.4%,** primary caregivers **89.5%**.

| Strongly disagree (1) | Disagree (2) | Neutral (3) | Agree (4) | Strongly agree (5) |
| --- | --- | --- | --- | --- |
|  |  |  |  |  |

1. Solid guidance with clear rules and structures is indispensable.

In Round 2: scientists **69.3%,** professional caregivers **61.9%,** primary caregivers **84.2%**.

| Strongly disagree (1) | Disagree (2) | Neutral (3) | Agree (4) | Strongly agree (5) |
| --- | --- | --- | --- | --- |
|  |  |  |  |  |

1. The assistance must always be accessible and available.

In Round 2: scientists **61.6%,** professional caregivers **66.7%,** primary caregivers **84.2%**.

| Strongly disagree (1) | Disagree (2) | Neutral (3) | Agree (4) | Strongly agree (5) |
| --- | --- | --- | --- | --- |
|  |  |  |  |  |

1. Mistakes can be made during the support process in order to learn.

In Round 2: scientists **84.6%,** professional caregivers **80.9%,** primary caregivers **73.7%**.

| Strongly disagree (1) | Disagree (2) | Neutral (3) | Agree (4) | Strongly agree (5) |
| --- | --- | --- | --- | --- |
|  |  |  |  |  |

1. Treatment by a multidisciplinary, ambulant team (FACT) is required.

In Round 2: scientists **77%,** professional caregivers **80.9%,** primary caregivers **73.7%**.

| Strongly disagree (1) | Disagree (2) | Neutral (3) | Agree (4) | Strongly agree (5) |
| --- | --- | --- | --- | --- |
|  |  |  |  |  |

1. Wrap-around care is required.

In Round 2: scientists **92.3%,** professional caregivers **76.2%,** primary caregivers **57.9%**.

| Strongly disagree (1) | Disagree (2) | Neutral (3) | Agree (4) | Strongly agree (5) |
| --- | --- | --- | --- | --- |
|  |  |  |  |  |

1. Different forms of treatment are required to eliminate the causes of problems experienced by individuals.

In Round 2: scientists **84.6%,** professional caregivers **66.7%,** primary caregivers **68.4%**.

| Strongly disagree (1) | Disagree (2) | Neutral (3) | Agree (4) | Strongly agree (5) |
| --- | --- | --- | --- | --- |
|  |  |  |  |  |

1. Case management is necessary in which support and treatment are aligned.

In Round 2: scientists **92.3%,** professional caregivers **95.3%,** primary caregivers **78.9%**.

| Strongly disagree (1) | Disagree (2) | Neutral (3) | Agree (4) | Strongly agree (5) |
| --- | --- | --- | --- | --- |
|  |  |  |  |  |

**Profile description**

**Profile 5:** **Persons with addictive problems**

Most people in this profile have borderline intellectual functioning and behavioural problems, and all are addicted to alcohol and/or drugs. This profile has an almost equal distribution between men and women; the average age is 28 years. More than half of these persons have no permanent residence or home. No-one in this profile has any form of daytime activities or work. Also, most of them have difficulty establishing relationships with their peers. A relatively large proportion has contact with the criminal world and/or contact with the police and judicial authorities. Debts are common. Most persons in this profile have a partner, and some have one or more children. Generally, the family in which they were raised had various problems; most of their parents are divorced. Many persons in this group were abused by their parents. In addition, many of the parents had debts, mental health problems, and also had difficulty raising their children in a consistent and appropriate way.

**Statements for persons in this profile:**

We would like to ask you again to indicate to what extent you agree with these statements.
For each statement you will see the percentages (pooled ‘Agree’ and ‘Strongly agree’) per expert group that emerged from Round 2.

For persons in this profile:

1. Housing is the main priority.

In Round 2: scientists **69.2%,** professional caregivers **90.4%,** primary caregivers **63.2%**.

| Strongly disagree (1) | Disagree (2) | Neutral (3) | Agree (4) | Strongly agree (5) |
| --- | --- | --- | --- | --- |
|  |  |  |  |  |

1. Realising a consistent and regular day structure is essential.

In Round 2: scientists **84.7%,** professional caregivers **76.2%,** primary caregivers **89.5%.**

| Strongly disagree (1) | Disagree (2) | Neutral (3) | Agree (4) | Strongly agree (5) |
| --- | --- | --- | --- | --- |
|  |  |  |  |  |

1. Attention for criminal behaviour is necessary.

In Round 2: scientists **76.9%,** professional caregivers **90.5%,** primary caregivers **89.5%.**

| Strongly disagree (1) | Disagree (2) | Neutral (3) | Agree (4) | Strongly agree (5) |
| --- | --- | --- | --- | --- |
|  |  |  |  |  |

1. Support should include the whole network (family, parents, partner, and own children).

In Round 2: scientists **92.4%,** professional caregivers **76.2%,** primary caregivers **84.2%.**

| Strongly disagree (1) | Disagree (2) | Neutral (3) | Agree (4) | Strongly agree (5) |
| --- | --- | --- | --- | --- |
|  |  |  |  |  |

1. Treatment of problem behaviour is indispensable.

In Round 2: scientists **69.2%,** professional caregivers **71.4%,** primary caregivers **84.2%**.

| Strongly disagree (1) | Disagree (2) | Neutral (3) | Agree (4) | Strongly agree (5) |
| --- | --- | --- | --- | --- |
|  |  |  |  |  |

1. Treatment by a multidisciplinary ambulant team (FACT) is required.

In Round 2: scientists **77%,** professional caregivers **85.7%,** primary caregivers **79%**.

| Strongly disagree (1) | Disagree (2) | Neutral (3) | Agree (4) | Strongly agree (5) |
| --- | --- | --- | --- | --- |
|  |  |  |  |  |

1. Wrap-around care is required.

In Round 2: scientists **84.6%,** professional caregivers **52.4%,** primary caregivers **73.7%.**

| Strongly disagree (1) | Disagree (2) | Neutral (3) | Agree (4) | Strongly agree (5) |
| --- | --- | --- | --- | --- |
|  |  |  |  |  |

1. Different forms of treatment are necessary to eliminate the causes of problems experienced by individuals.

In Round 2: scientists **84.6%,** professional caregivers **61.9%,** primary caregivers **79%.**

| Strongly disagree (1) | Disagree (2) | Neutral (3) | Agree (4) | Strongly agree (5) |
| --- | --- | --- | --- | --- |
|  |  |  |  |  |

1. One entrance to different forms of treatment and support is essential.

In Round 2: scientists **61.6%,** professional caregivers **81%,** primary caregivers **100%.**

| Strongly disagree (1) | Disagree (2) | Neutral (3) | Agree (4) | Strongly agree (5) |
| --- | --- | --- | --- | --- |
|  |  |  |  |  |

1. More funds are required from government for treatment and support.

In Round 2: scientists **76.9%,** professional caregivers **85.7%,** primary caregivers **78.9%.**

| Strongly disagree (1) | Disagree (2) | Neutral (3) | Agree (4) | Strongly agree (5) |
| --- | --- | --- | --- | --- |
|  |  |  |  |  |
